# Enhancing Photoelectrochemical Water Oxidation Using Ferromagnetic Materials and Magnetic Fields

**DOI:** 10.1021/jacs.4c13017

**Published:** 2024-12-04

**Authors:** Qingjie Wang, Louise I. Oldham, Alfredo Giner-Requena, Zeyuan Wang, Daniele Benetti, Salvador Montilla-Verdú, Rong Chen, Dongfeng Du, Teresa Lana-Villarreal, Ulrich Aschauer, Néstor Guijarro, James Robert Durrant, Jingshan Luo

**Affiliations:** † Institute of Photoelectronic Thin Film Devices and Technology, State Key Laboratory of Photovoltaic Materials and Cells, Tianjin Key Laboratory of Efficient Solar Energy Utilization, Ministry of Education Engineering Research Center of Thin Film Photoelectronic Technology, 12538 Nankai University, Tianjin 300350, China; ‡ Institute of Electrochemistry, 16718 Universidad de Alicante, Apartat 99, E-03080 Alacant, Spain; § Centre for Processable Electronics, Department of Chemistry, 4615 Imperial College London, London SW7 2AZ, U.K.; ∥ Department of Chemistry and Physics of Materials, 625366 University of Salzburg, Jakob-Haringer-Str. 2A, Salzburg 5020, Austria; ⊥ Frontiers Science Center for New Organic Matter, Nankai University, Tianjin 300071, China; # Haihe Laboratory of Sustainable Chemical Transformations, Tianjin 300192, China

## Abstract

Photoelectrochemical (PEC) water splitting provides a promising strategy for H_2_ production. However, its performance is limited by severe carrier recombination and sluggish water oxidation kinetics. While numerous strategies, namely, elemental doping, morphology engineering, heterojunction formation, and catalyst modification, have been extensively explored to enhance the PEC performance, the application of external magnetic fields (MFs) to affect the catalysis or charge carrier dynamics remains yet to be exploited. Herein, BiVO_4_ is first selected as a representative photoanode, demonstrating that an ultrathin ferromagnetic coating based on Fe_2_TiO_5_, when combined with an external MF, boosts its solar water oxidation performance. The combined analyses of the charge transfer and separation efficiency together with ultraviolet photoelectron spectroscopy and transient absorption spectroscopy data revealed that the MF positively affects the band alignment across the BiVO_4_/Fe_2_TiO_5_ interface, improving the charge separation, while the oxygen evolution at the Fe_2_TiO_5_/electrolyte interface was promoted. Finally, we expand this concept to other metal oxide photoanodes, such as TiO_2_, WO_3_, and Fe_2_O_3_, demonstrating the universality of such an approach. Overall, this work pioneers a novel route to harvest external MFs and improve the PEC response of common nonmagnetic semiconductor photoelectrodes in photoelectrocatalytic conversion.

## Graphical abstract

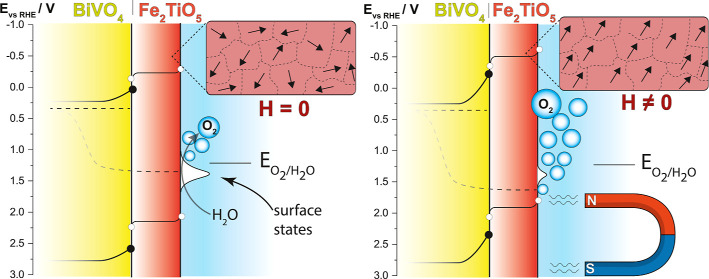

## Introduction

Photoelectrochemical (PEC) water splitting emerged as a promising strategy for converting solar energy into hydrogen.^1–7^ One of the major bottlenecks encountered in achieving high-performance PEC devices originates from the semiconductor photoanode and the high-energy-demanding oxygen evolution reaction (OER). Over the past decade, several strategies have been explored to improve the photoanodes’ activity toward the OER, namely, elemental doping,^8,9^ morphology control,^10^ heterojunction construction,^11,12^ coupling catalysts,^13,14^ etc. However, despite these efforts, the overall efficiency of the device remains insufficient for practical applications, and therefore, new strategies to manipulate the photogenerated charge carrier dynamics and improve the interfacial charge transfer are demanded.

Interestingly, in electrocatalytic systems for the OER, the application of external magnetic fields (MFs) was demonstrated to boost the performance. For example, Xu et al. reported that using ferromagnetic ordered CoFe_2_O_4_ catalysts as spin polarizers enhanced the OER under a constant MF.^15^ Liu et al. demonstrated a spin-dependent reaction pathway using spin-rearranged Co_0.8_Mn_0.2_ MOF, resulting in excellent electrocatalytic OER activity due to spin-correlated catalytic activity.^16^ Likewise, Xu et al. revealed that magnetizing a ferromagnetic material changed its magnetic domain structure, improving mass transport and accelerating reaction kinetics.^17^ Based on these encouraging results, although scarce, some authors have studied the impact of MF on the PEC performance of semiconductor photoelectrodes. Sang and co-workers demonstrated that applying a MF to the ferromagnetic ZnFe_2_O_4_ photoanode accelerated its charge separation process. The authors argued that the external MF modulated the spin polarization, affecting the magnetoresistance and the recombination, resulting in an improved PEC performance.^18^ Likewise, Chen et al. demonstrated that the performance of FeCoSe_2_ photoanodes is enhanced under an applied MF primarily due to the reduced charge recombination and the improved catalytic activity toward OER.^19^ To date, however, the use of MFs to enhance the performance of semiconductor photoelectrodes has been limited to ferromagnetic materials, drastically reducing the family of materials that could benefit from such an approach. Note that some studies have reported improved PEC performance of nonmagnetic photoelectrodes under applied MFs.^20^ However, physical phenomena related to the magnetohydrodynamics and the improved mass transfer in the electrolyte could be invoked to explain the improved performance in these cases rather than any impact on the charge carrier dynamics or the catalysis at the photoelectrode. Therefore, enabling new strategies to leverage the benefits of MF in conventional semiconductor photoelectrodes is crucial to advancing the field of PEC water oxidation.

Inspired by these works, we propose a general strategy to improve PEC water oxidation performance of conventional nonmagnetic photoelectrodes by coupling with a ferromagnetic material and applying an external MF. To make it work, the ferromagnetic materials should have a good band alignment with the photoelectrode materials and should exhibit a positive effect under the magnetic stimulus. Herein, we first selected BiVO_4_ as a representative photoanode to demonstrate the concept by coating it with Fe_2_TiO_5_. The composite BiVO_4_/Fe_2_TiO_5_ photoelectrode shows enhanced PEC water oxidation performance due to the formation of a heterojunction and improved catalytic activity. Additionally, applying an external MF further improves its PEC response from 3.06 to 3.33 mA/cm^2^ at 1.23 V vs RHE. A comprehensive analysis revealed that the MF improved not only the catalytic response but also the charge separation by promoting the spin polarization and the adjustment of the energy bands at the heterojunction, respectively. Furthermore, we extend this concept to other promising photoanodes, such as TiO_2_, WO_3_, and Fe_2_O_3_, showing its universality. Overall, this work pioneers a new strategy to “sensitize” conventional nonmagnetic semiconductor materials to MFs, leveraging in such a way their beneficial effects over the performance of PEC devices.

## Results and Discussion

BiVO_4_ was fabricated via a two-step method involving electrodeposition followed by annealing in air, whereas Fe_2_TiO_5_ was grown using a hydrothermal method followed by annealing. Detailed procedures are available in the Supporting Information. Fe_2_TiO_5_ is a chemically robust n-type narrow band gap ferromagnetic photoanode, crystallizing in an orthorhombic structure (Figure S1).^21–23^ X-ray diffraction (XRD) patterns of BiVO_4_, Fe_2_TiO_5_, and BiVO_4_/Fe_2_TiO_5_ thin films are shown in Figure 1a. Notably, the incorporation of Fe_2_TiO_5_ did not alter the XRD pattern of pristine BiVO_4_ likely due to the ultrathin nature of the coating. UV–vis spectra of BiVO_4_ and Fe_2_TiO_5_ thin films revealed absorption onset values ca. 500 and 580 nm, respectively (Figure S2), consistent with the reported band gaps of 2.5 and 2.17 eV.^13,22,24–26^ X-ray photoelectron spectroscopy (XPS) was utilized to analyze the chemical environment and the oxidation states in both pristine and Fe_2_TiO_5_-coated BiVO_4_ thin films. Notably, the Bi 4f and V 2p spectra exhibited a slight shift (0.15 eV) toward lower binding energies upon Fe_2_TiO_5_ coating (Figure 1b,c), indicating a strong interaction between BiVO_4_ and Fe_2_TiO_5_. The corresponding O 1s shows drastic changes likely related to the fact that the oxygen environments on Fe_2_TiO_5_ and BiVO_4_ are different (Figure 1d). Finally, the Fe 2p and Ti 2p XPS spectra corresponding to the heterostructure shown in Figure 1e, confirm the formation of the BiVO_4_/Fe_2_TiO_5_ heterojunction. Scanning electron microscopy (SEM) revealed that pristine BiVO_4_ thin films exhibited a worm-like morphology with an average thickness of ca. 1 μm (Figure S3), while Fe_2_TiO_5_ deposition increased the surface roughness (Figure 1f,g). Spherical aberration correction high-resolution transmission electron microscopy (HRTEM) imaging revealed an amorphous 4 nm-thick layer uniformly coating the surface of BiVO_4_, which was identified by the lattice spacing of 0.467 and 0.292 nm, characteristic of the (011) and (040) planes of monoclinic BiVO_4_ (Figure 1h). Notably, high-angle annular dark-field scanning TEM (HAADF-STEM) and energy-dispersive X-ray spectroscopy (EDX) mapping showed that Fe_2_TiO_5_ was homogeneously distributed over the BiVO_4_ particles (Figure 1i–m). Further analysis confirmed that the Fe/Ti atomic ratio was 1.58:0.85 (Figure S4), i.e., matching the nominal value of 2:1.

**Figure 1:**
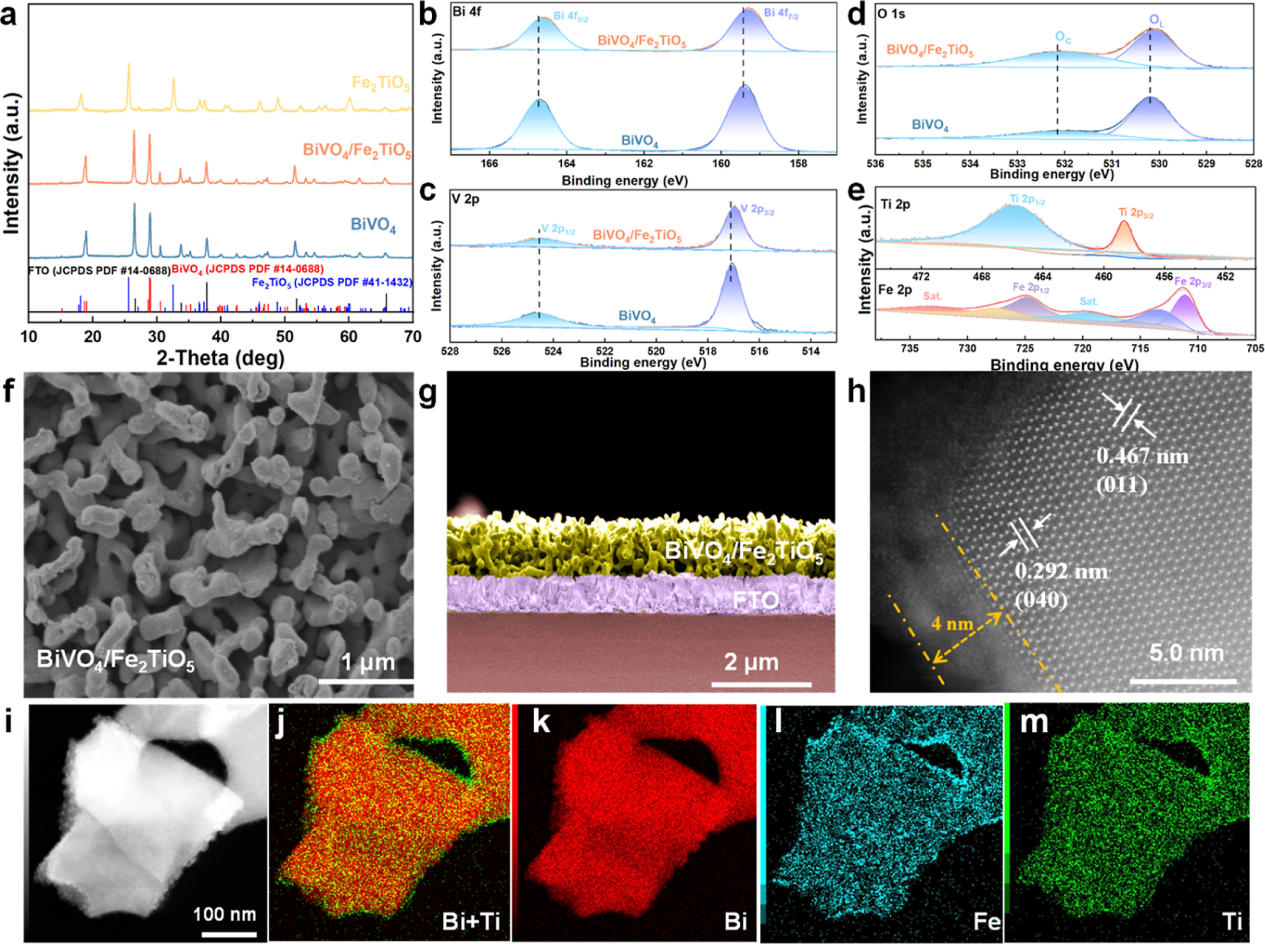
Structural properties of the BiVO_4_/Fe_2_TiO_5_ thin film. (a) XRD patterns of BiVO_4_, Fe_2_TiO_5_, and BiVO_4_/Fe_2_TiO_5_. (b–e) High-resolution XPS of Bi 2p, V 2p, O 1s, Ti 2p, and Fe 2p elements. (f) Top-view SEM and (g) cross-section SEM images of the BiVO_4_/Fe_2_TiO_5_ photoanode. (h) Spherical aberration correction HRTEM image, (i–m) HAADF-STEM image of the BiVO_4_/Fe_2_TiO_5_ photoanode and EDX elemental mapping images for Bi + Ti, Bi, Fe, and Ti, respectively.

The energy-level positions of BiVO_4_ and Fe_2_TiO_5_ were estimated by using ultraviolet photoelectron spectroscopy (UPS) measurements (Figure S2d). The schematic energy band structure of BiVO_4_ and Fe_2_TiO_5_ exhibits a staggered profile, i.e., a type-II heterojunction (Figure 2a), which is anticipated to effectively promote charge separation. The PEC response of both BiVO_4_ and BiVO_4_/Fe_2_TiO_5_ photoanodes was recorded under simulated (AM 1.5G) solar illumination. Figure 2b shows that the photocurrent recorded at 1.23 V vs RHE increased from 1.64 (BiVO_4_) to 3.03 mA/cm^2^ upon coating with Fe_2_TiO_5_. In addition, a cathodic shift in the onset potential from 0.5 to 0.35 V_RHE_ was detected. The incident photon-to-current conversion efficiency (IPCE) profiles correlate well with the absorption spectra. IPCE values for the BiVO_4_/Fe_2_TiO_5_ photoanode are 50% ∼ 60% from 350 to 450 nm, ca. 30% higher than that of pristine BiVO_4_ (Figure S5). The integrated photocurrents are 1.62 and 3.2 mA/cm^2^ for BiVO_4_ and BiVO_4_/Fe_2_TiO_5_, respectively, consistent with the values obtained from the *J*–*V* curves (Figure S6).

**Figure 2:**
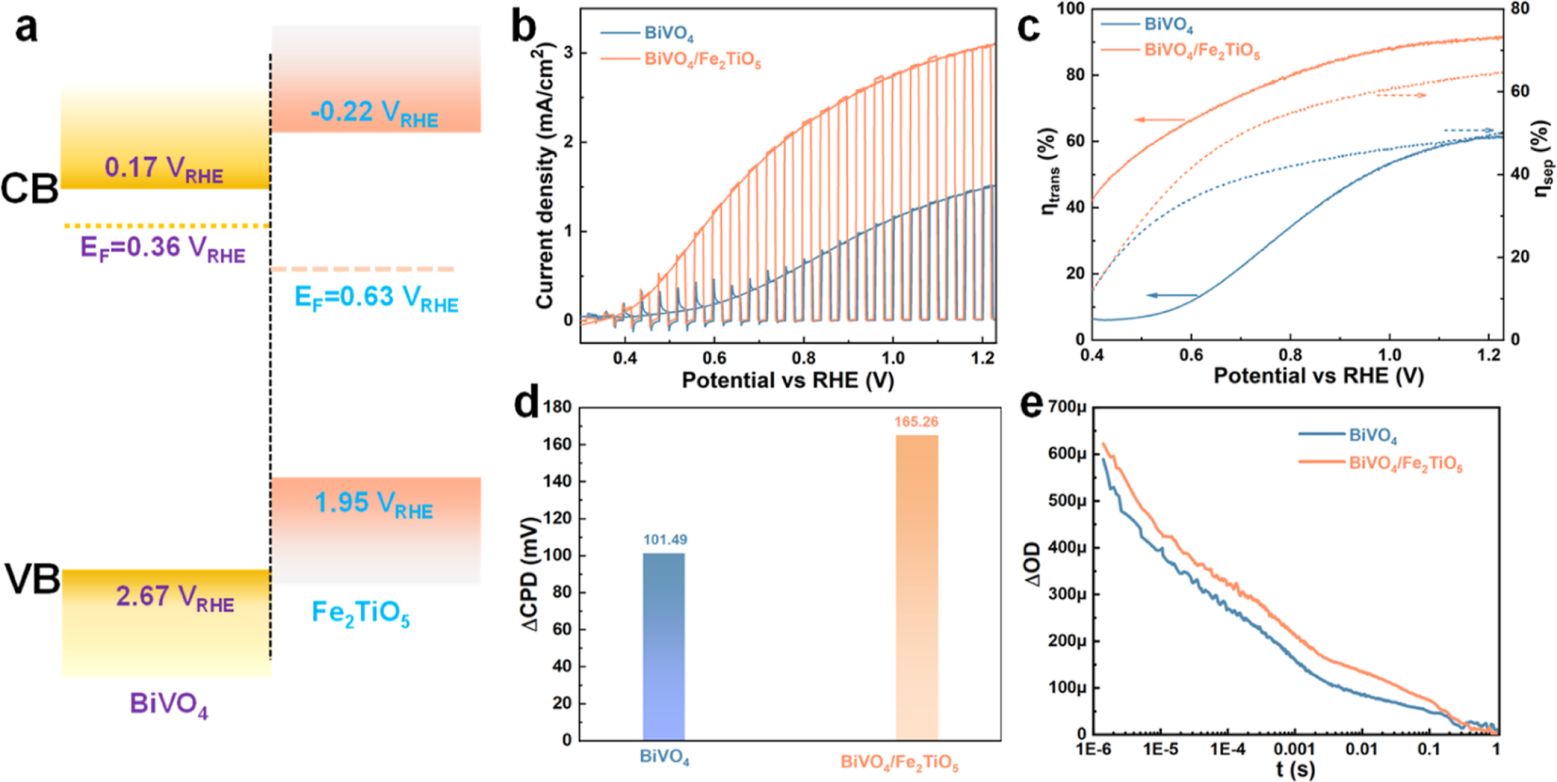
PEC properties. (a) Calculated energy band structure of BiVO_4_ and Fe_2_TiO_5_, (b) *J*–*V* curves of BiVO_4_ and BiVO_4_/Fe_2_TiO_5_ photoanode, (c) charge separation efficiency (η_sep_, right dashed line) and charge transfer efficiency (η_trans_, left solid line) of the BiVO_4_/Fe_2_TiO_5_ photoanode, and (d) calculated ΔCPD of BiVO_4_ and BiVO_4_/Fe_2_TiO_5_ photoanode. (e) Transient absorption decays of BiVO_4_ (blue) and BiVO_4_/Fe_2_TiO_5_ (orange–red) under an applied bias of 1.2 V_RHE_. Decay traces probed at 700 nm after front-side laser excitation at 355 nm (∼300 μJ cm^–2^, 1 Hz). Measurements were carried out in 1 M KBi buffer (pH 9.5).

To elucidate the impact of surface treatments on the bulk and surface properties, the charge separation (η_sep_) and transfer efficiency (η_trans_) were estimated (Figure S7a). The η_sep_ improved from 50.3% to 64.7% at 1.23 V vs RHE upon deposition of Fe_2_TiO_5_ (Figure 2c, dash line). This could be attributed to the improved charge separation resulting from the heterojunction formation. Notably, coating with Fe_2_TiO_5_ caused a significant increase in η_trans_ from 70% to 91% at 1.23 V vs RHE (solid line), attributed to the enhanced surface catalysis toward the OER. Previous studies have indicated that Fe_2_TiO_5_ acts as an efficient electrocatalyst, facilitating charge transfer to the electrolyte.^27–29^ Both the improved η_sep_ and η_trans_ account for the enhanced photocurrent and earlier onset detected in Figure 2b. Dark *J*–*V* curves of the BiVO_4_ and Fe_2_TiO_5_ electrodes are displayed in Figure S8. Here, the onset potential of Fe_2_TiO_5_ shifted toward more negative potentials compared to BiVO_4_ supporting the improved OER activity of the former.^27,30,31^ Additionally, Kelvin probe force microscopy (KPFM) was employed to spatially resolve the potential distribution on the BiVO_4_ electrode’s surface (Figure S9), under both dark and light irradiation. The surface potentials of all photoanodes decreased significantly under light irradiation, suggesting the accumulation of photogenerated holes on the surface. Moreover, the average surface photovoltage (ΔCPD, Figure 2d) of BiVO_4_/Fe_2_TiO_5_ was approximately 65 mV higher than that of BiVO_4_, supporting the improved charge separation at the BiVO_4_/Fe_2_TiO_5_ interface. Likewise, open-circuit potential (OCP) measurements showed that under illumination, the OCP values shifted cathodically for the BiVO_4_/Fe_2_TiO_5_ photoanode (Figure S7b). This provided evidence of mitigation of the intrinsic Fermi level pinning occurring on BiVO_4_ when Fe_2_TiO_5_ is deposited. Note as well that the measured ΔOCP (ΔOCP = *E*_OCP_^dark^ – *E*_OCP_^light^) values for BiVO_4_ and BiVO_4_/Fe_2_TiO_5_ photoanode were 143.3 and 204.6 mV, respectively (Figure S7c), indicating a more favorable driving force for PEC water oxidation.^2,26,32^ Notably, the OCP under illumination shifted to 278.1 mV for the BiVO_4_/Fe_2_TiO_5_ photoanode, further supporting the role of Fe_2_TiO_5_ at promoting charge separation and facilitating efficient water oxidation.

Transient absorption spectroscopy (TAS) was employed to directly probe the population and lifetimes of photoinduced carriers in both bare and bipolarized BiVO_4_/Fe_2_TiO_5_ photoanodes. Figure 2e shows the change in optical density (ΔOD) of BiVO_4_ and BiVO_4_/Fe_2_TiO_5_ upon excitation over a μs–s time scale at an applied bias of +1.2 V vs RHE (see Figure S11 for measurements as a function of the applied bias). Previous TAS studies have shown that photogenerated holes in BiVO_4_ show a broad absorption feature peaking at 550 nm.^33^ In this study, probe wavelengths of 700 and 650 nm were selected to investigate BiVO_4_ hole populations as the TAS spectra (Figure S10) obtained for these samples showed a flat absorption feature, and these slightly longer wavelengths gave significantly improved signal-to-noise compared to 550 nm. The TAS decays in Figure 2e show clear biphasic behavior, with the decays at earlier timescales (μs–ms) assigned to bulk recombination and with those at later timescales (ms–s) assigned to the competition between back-electron recombination and water oxidation.^34^ Note that, as expected, these two decay regimes become more distinct at a higher anodic bias (Figure S11). The bare BiVO_4_ and BiVO_4_/Fe_2_TiO_5_ films both showed increased hole-related signals under higher anodic bias (Figure S11a,b) due to the increased band bending, in agreement with previous studies on metal oxides, including BiVO_4_.^11^ It is worth highlighting that the Fe_2_TiO_5_ coating enhanced the magnitude of the ΔOD signal at all timescales, indicating a higher density of photogenerated holes. This confirms that the addition of Fe_2_TiO_5_ improves charge separation, and it is consistent with the formation of a type-II heterojunction.^11^ The increased hole population is particularly significant on the timescales relevant to water oxidation and under higher applied bias (Figure S11c).

The magnetic properties of Fe_2_TiO_5_ powder were characterized by measuring the magnetization (*M*) as a function of the MF (*H*). Figure 3a portrays its room-temperature ferromagnetic behavior,^21^ with the *M*–*H* curve exhibiting significant coercivity (−24.62 Oe) and remanence (2.54 × 10^–4^ emu/g) across the range from −400 to 400 Oe. Further investigation of the Fe_2_TiO_5_ thin film using atomic force microscopy (AFM) did not show significant changes upon applying an external MF (Figure 3b,c top). However, magnetic force microscopy (MFM) evidenced clear alterations in the magnetic domain pattern after applying MF. Without MF, distinct magnetic domains (red and blue regions, Figure 3d) indicated well-separated magnetic phases (opposite polarity).^17^ With the MF, the magnetic domains of Fe_2_TiO_5_ aligned, transitioning into a nearly single-domain configuration (Figure 3e). This alignment phenomenon further confirmed the ferromagnetic properties of the Fe_2_TiO_5_ thin film.

**Figure 3:**
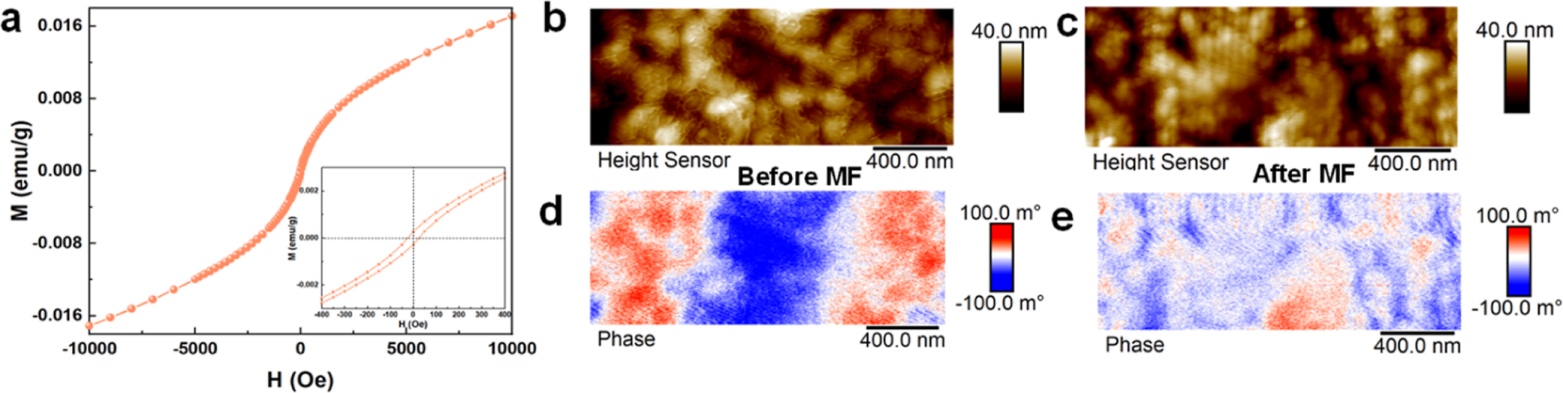
(a) *M*–*H* curves of Fe_2_TiO_5_. Topographic (AFM, top) images of Fe_2_TiO_5_ films: (b) unmagnetized and (c) magnetized under an out-of-plane MF of 2000 Oe. Zero-field MFM phase (bottom) images of Fe_2_TiO_5_ films: (d) unmagnetized and (e) magnetized under an out-of-plane MF of 2000 Oe.

Next, the influence of the MF on the PEC performance of the BiVO_4_/Fe_2_TiO_5_ heterojunction was investigated by including a magnet near the photoanode in a three-electrode configuration (Figures 4a and S15). The *J*–*V* curves depicted in Figures 4b and S12 demonstrated that the performance of BiVO_4_/Fe_2_TiO_5_ photoanodes significantly improved under the influence of the MF, whereas that of pristine BiVO_4_ barely changed. This corroborated the notion that the improved performance stems from the interaction of Fe_2_TiO_5_ with the applied MF. At 1.23 V vs RHE, the photocurrent density increased from 3.06 to 3.33 mA/cm^2^ with the incorporation of the MF. Moreover, increasing the MF intensity resulted in a proportional increase of the photocurrent at 1.23 V_RHE_, highlighting, thus, the direct influence of the MF strength on the *J*–*V* response (Figure S13). Transient magnetic field-induced photocurrent measurements (Figure 4c) further revealed that BiVO_4_/Fe_2_TiO_5_ exhibited an immediate positive response to the MF application (A-MF: applying MF, R-MF: removing MF), contributing to approximately a 7.3% increase in photocurrent at 1.23 V vs RHE. Note that the reflection of light at the magnet was ruled out to be contributing to the enhanced PEC response by using an antireflective coating (Figure S14).

**Figure 4:**
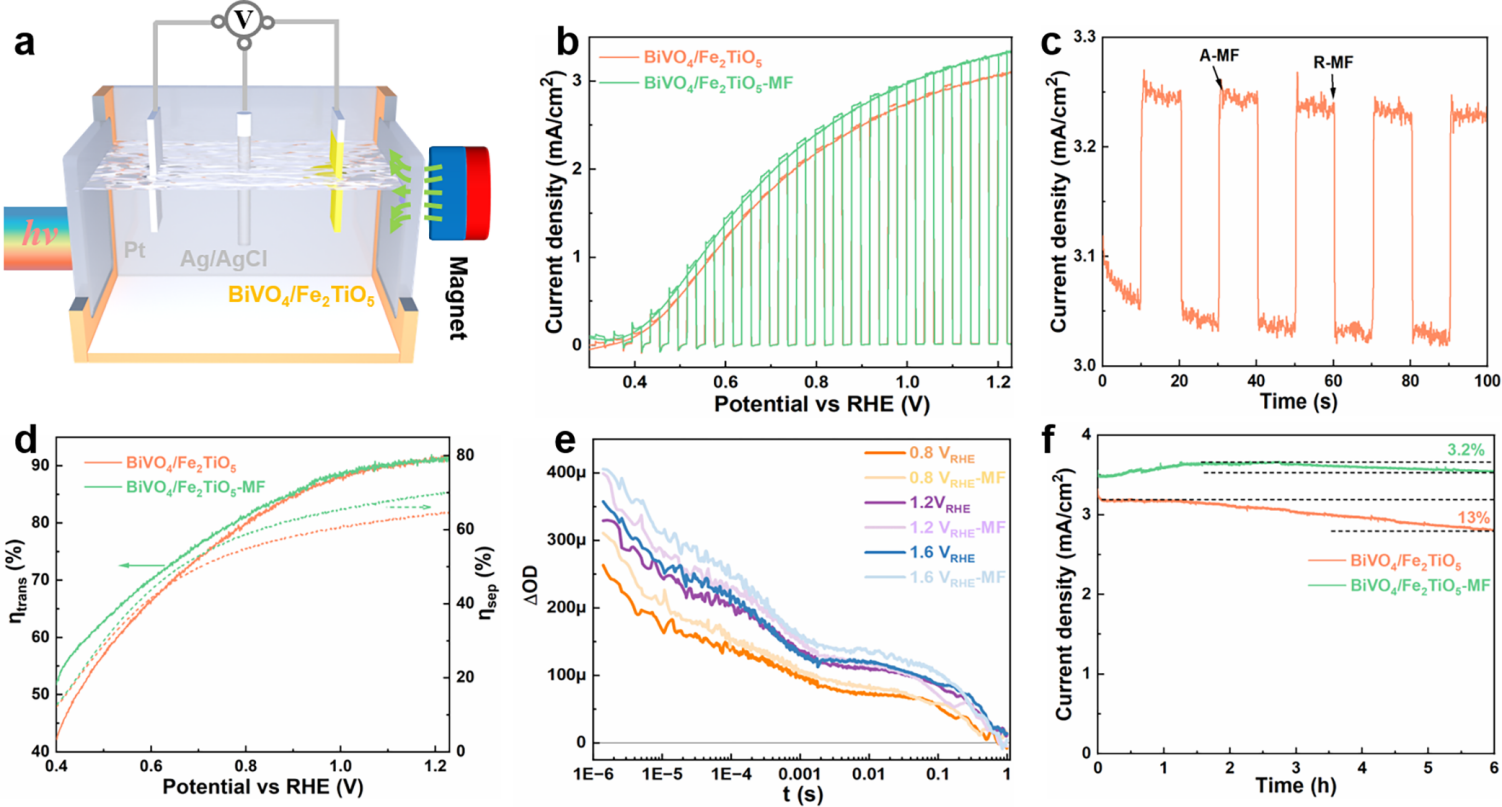
(a) Schematic diagram of the experimental setup of the MF-enhanced PEC system. (b) *J*–*V* curves recorded for a BiVO_4_/Fe_2_TiO_5_ photoanode first in the absence and, second, in the presence of the MF under AM 1.5 G illumination. (c) Transient magnetic response curves of a BiVO_4_/Fe_2_TiO_5_ photoanode recorded at 1.23 V vs RHE. (d) The η_trans_ (left solid line) and η_sep_ (right dashed line) values of BiVO_4_/Fe_2_TiO_5_ with or without MF. (e) Transient absorption decays of a BiVO_4_/Fe_2_TiO_5_ recorded at an applied bias of 1.2 V_RHE_, recorded before (orange) and after (purple) exposure to an external MF. For the magnetization process, the magnet was placed in front of the sample for 1.5 h and then removed before obtaining the second TAS trace. Decay traces probed at 650 nm after back-side laser excitation at 355 nm (∼300 μJ cm^–2^, 1 Hz). Measurements were carried out in 1 M KBi buffer, pH 9.5. MF = magnetic field. (f) Stability test performed by recording the photocurrent at 1.23 V vs RHE of two different samples of BiVO_4_/Fe_2_TiO_5_, one in the absence and another one in the presence of an external MF.

Once the positive effect of the MF on the PEC response was established, the η_sep_ and η_trans_ values together with fast-scan cyclic voltammetry were gathered to rationalize the role of the MF. The η_trans_ of BiVO_4_/Fe_2_TiO_5_ increases at potentials below 1.0 V vs RHE under an external MF stimulus. This could be attributed to an improved surface passivation and catalytic activity triggered by the MF (Figure 4d). To explore the former phenomenon, fast-scan cyclic voltammetry experiments were performed (Figure S17). As observed, the cathodic peak located at around 1.5 V vs RHE, typically ascribed to the reduction of trapped holes at the interface and, therefore, to the surface states recombination (r-ss),^35,36^ decreased in the case of BiVO_4_/Fe_2_TiO_5_ when the MF was incorporated. Quantitatively, the accumulated charge (*Q*_r‑SS_) was measured to be 0.015 C for BiVO_4_, 0.012 C for BiVO_4_/Fe_2_TiO_5_, and 0.01 C for BiVO_4_/Fe_2_TiO_5_-MF (Figure S17c). The improved catalytic activity of Fe_2_TiO_5_ toward the OER was evidenced by the earlier onset and reduced resistance for charge transfer exhibited by the Fe_2_TiO_5_ electrodes when performing linear sweep voltammograms (Figure S8). Overall, this suggests that the application of MF effectively mitigates surface recombination processes and accelerates the OER, leading to improved charge separation and transfer dynamics and, therefore, enhanced PEC performance.

In addition, η_sep_ was further enhanced at high applied bias for BiVO_4_/Fe_2_TiO_5_ when the MF was incorporated, indicating that the MF effectively promotes charge separation (Figure 4d). This improvement can be primarily attributed to two factors. First, the decreased resistance of Fe_2_TiO_5_ under the external MF, as evidenced by electrochemical impedance spectroscopy (Figure S8). While the resistance remained unchanged for BiVO_4_ with/without MF, the charge transfer resistance significantly decreased for Fe_2_TiO_5_ with MF. The fact that the charge recombination is suppressed is also observed in other ferromagnetic semiconductors, analogous to the giant magnetoresistance effect.^37,38^ Second, the improved η_sep_ may result from the change in the band alignment. After applying the MF, the Fermi level of Fe_2_TiO_5_ was found to shift upward by UPS analysis (Figure S18), in agreement with the shift of the flat band detected by means of the Mott–Schottky plot (Figure S19). This reinforces the notion that the built-in electric field is strengthened under the MF, resulting in a larger photovoltage. This is further supported by the enhanced photocurrent recorded in the presence of a sacrificial hole scavenger (Figure S12c). However, to the best of our knowledge, there is currently no theoretical explanation for the MF-induced shift of the Fermi level. In nonmagnetic semiconductors, the density of states (DOS) is evenly distributed between spin-up and spin-down states, whereas in ferromagnetic semiconductors, one spin direction is favored due to alignment with the magnetization, leading to an “exchange splitting energy” between majority and minority spin states (Figure 5a,b).^18,39^ Bearing this in mind, we hypothesize that after the magnetization of Fe_2_TiO_5_ the distribution of DOS for spin-up and spin-down electrons changed, therefore altering the resulting Fermi level. The resulting preferential spin polarization of holes within the more uniform and larger ferromagnetic domains could preferentially favor the occurrence of the OER via the triplet O_2_ ^40,41^ and therefore explain the improved catalytic response.

**Figure 5:**
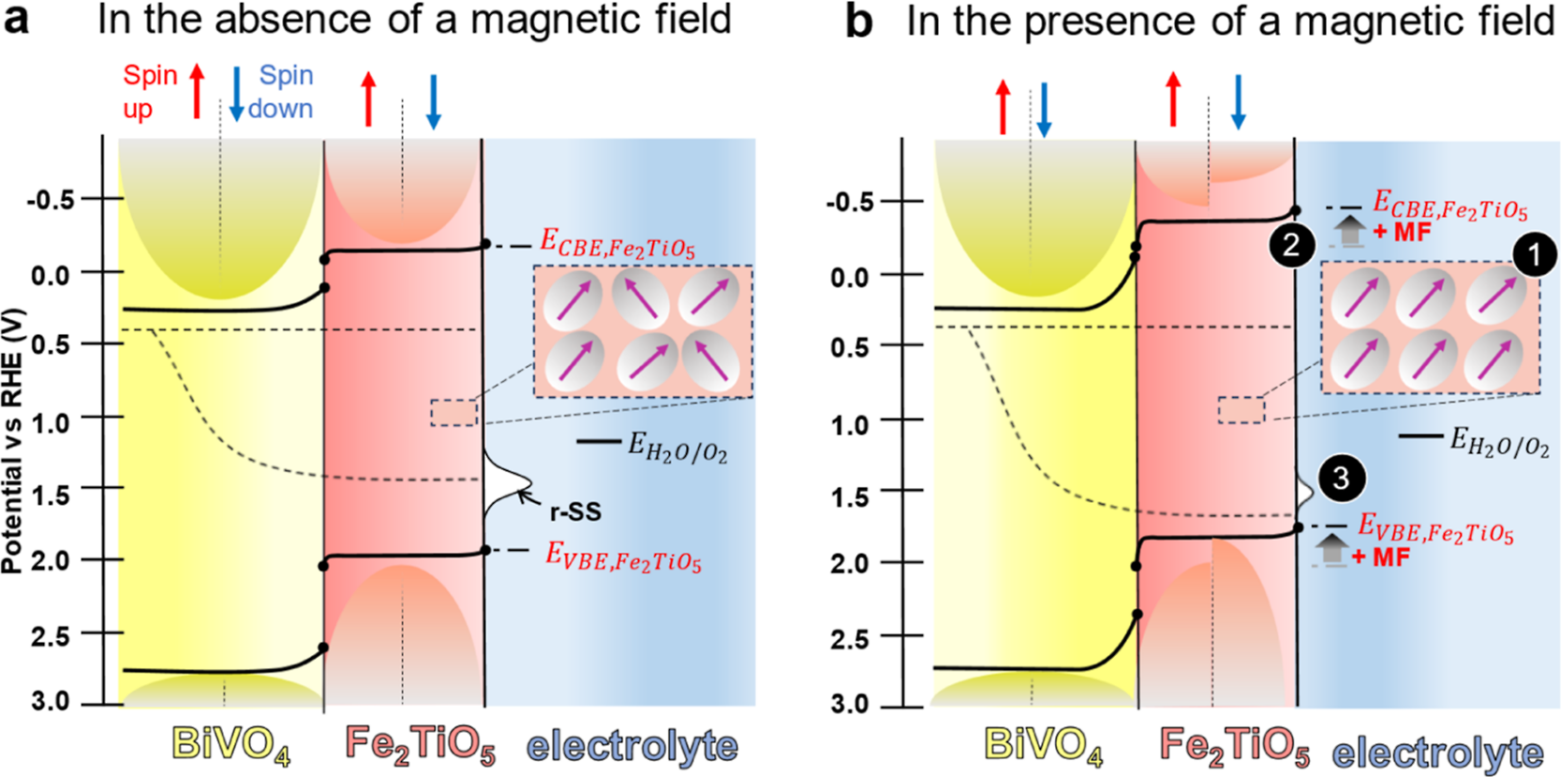
Energy-level diagram of BiVO_4_ and Fe_2_TiO_5_ portraying the alignment of the magnetic domains (1), the change in the energy of the conduction band edge (*E*_CBE_) and of the valence band edge (*E*_VBE_) (2), and the reduced density of r-SS (3) both in the absence (a) and in the presence (b) of an external MF. A scheme of the DOS of both semiconductors as a function of the potential indicating the spin-up and spin-down contributions is included to portray the proposed hypothesis whereby the band shift observed on the Fe_2_TiO_5_ sample, under a MF, could originate from the changes in the DOS of the spin-up and -down. A cartoon depicting the alignment of the magnetic domains on the Fe_2_TiO_5_ is also included.

Figure 4e shows TAS measurements of BiVO_4_/Fe_2_TiO_5_ before and after exposure to an external MF. An increase in photocurrent density in BiVO_4_/Fe_2_TiO_5_ after exposure to a MF was correlated with an increase in hole population, particularly at earlier timescales. This suggests that the magnetization process reduces bulk recombination and improves the charge separation. This is in agreement with other characterization properties, including OCP (Figures S7 and S16) and MS spectra (Figure S19). Bias-dependent TAS measurements of both BiVO_4_ and BiVO_4_/Fe_2_TiO_5_ with and without MF are shown in Figure S21. The TAS decays of BiVO_4_ remained unchanged after the addition of the external MF at all applied biases, while the BiVO_4_/Fe_2_TiO_5_ hole signals increased in the presence of the MF. This demonstrates the benefits of an external MF in enhancing the performance beyond the improvement triggered by the heterojunction formation. Furthermore, the fact that the same behavior is not observed in the bare BiVO_4_ highlighted the role of ferromagnetic-dependent spin polarization and the parallel arrangement of electron spin polarization in enhancing charge separation and transfer efficiency.

The PEC stability is recorded in Figure 4f and illustrates that BiVO_4_/Fe_2_TiO_5_ shows a 13% loss of photocurrent after 6 h. Characterization of the Fe_2_TiO_5_-coated films after the stability test did not reveal changes in XRD, although slight morphological changes were observed (Figures S22 and S24). These changes could be ascribed to the partial photocorrosion of the material under operation. Note that the incorporation of MF drastically affects the stability. Indeed, the photocurrent density of BiVO_4_/Fe_2_TiO_5_ under the MF decreased only 3% after 6 h of irradiation. SEM images and XPS spectra confirmed the excellent stability of the BiVO_4_/Fe_2_TiO_5_ photoanode after testing with MF (Figures S24–S26), suggesting that MF enhances stability by accelerating charge separation and extraction and mitigating charge recombination. Note that the XPS data evidenced slight shifts in the characteristic peak positions after PEC testing that do not support drastic changes in the composition or in the oxidation states of the elements.

Collectively, these characterizations demonstrate that applying MFs to the BiVO_4_/Fe_2_TiO_5_ photoanode significantly enhanced the photocurrent (∼10%) driven by three synergistic factors that improve the PEC water oxidation performance (Figure 5a,b). (i) The fully aligned magnetic domain structure of Fe_2_TiO_5_ under MF facilitates spin-selective electron transfer, thereby improving the catalytic activity (Figure 3c); (ii) the upward shift of the conduction band edge (*E*_CBE_) and valence band edge (*E*_VBE_) of Fe_2_TiO_5_ strengthens the built-in electric field and the driving force for the hole transfer across the BiVO_4_/Fe_2_TiO_5_ interface, accelerating the charge separation (Figure S15); and (iii) the reduced density of r-SS alleviates the trapping of photogenerated holes at surface recombination centers, promoting hole injection into the electrolyte for participation in the water oxidation reaction, as demonstrated by the decrease of *Q*_r‑SS_ (Figure S17).

To illustrate the versatility of the strategy, three common nonmagnetic photoelectrodes, namely, TiO_2_, WO_3_, and α-Fe_2_O_3_, were employed in conjunction with Fe_2_TiO_5_, and their corresponding PEC water oxidation activity was evaluated (Figures S27–S29). The PEC performance of these photoelectrodes with or without MF is shown in Figure S30. Notably, for the WO_3_ photoanode, coating with Fe_2_TiO_5_ induced a cathodic shift in the onset potential from 0.8 to 0.6 V vs RHE, and the photocurrent increased by approximately ∼6% at 1.23 V vs RHE with magnetic stimulation (Figure S30a). Similarly, the TiO_2_/Fe_2_TiO_5_ photoanode showed a ∼14% improvement in photocurrent under MF stimulation, with the onset potential shifting cathodically from 0.3 to 0.2 V vs RHE (Figure S30b). The enhanced photocurrent response under MF can be attributed to the favorable interface contact and appropriate energy band alignment between TiO_2_ and Fe_2_TiO_5_ (Figures S31–S33). This trend is also observed in the PEC activity of the Fe_2_O_3_/Fe_2_TiO_5_ photoanode (Figure S30c). Furthermore, the larger built-in electric field among WO_3_, TiO_2_, and Fe_2_TiO_5_ enhances the driving force for accelerating carrier separation under MF conditions. Therefore, employing ferromagnetic semiconductors to modify nonmagnetic photoelectrodes proves to be a viable strategy for enhancing PEC activity under MF stimulation.

## Conclusions

In summary, our study proposes a versatile strategy to enhance PEC water oxidation activity using external MFs by combining conventional nonmagnetic semiconductors (BiVO_4_) with ferromagnetic overlayers (Fe_2_TiO_5_). Here, it was demonstrated that the presence of the MF improves the photocurrent density primarily owing to the alignment of the magnetic domains of the overlayer, which prompted the improved OER kinetics, the mitigation of the Fermi-level pinning, and, specially, an enhanced photogenerated charge separation. Interestingly, it was found that ferromagnetic materials in the presence of MFs were able to induce drastic changes in the band bending of underlying nonmagnetic materials, therefore boosting the charge separation. Although the mechanism for this phenomenon remains speculative and demands further investigation, understanding these intricacies of MF-induced enhancements is crucial to fully realizing their potential. Moreover, the use of magnetic stimulation not only provides new insights and impacts in the PEC field but also suggests potential applications and benefits across other related fields. Exploring MF effects opens exciting avenues for novel advancements and interdisciplinary collaborations aimed at leveraging magnetic properties for improved performance in various technological applications.

## Supplementary Material



## Notes

Experimental section; additional interpretations of some results; crystal structure of Fe_2_TiO_5_; UV–vis adsorption spectra; XPS and UPS tests; SEM, TEM, HRTEM, and EDX-line scanning images; IPCE curves and LSV curves for BiVO_4_ and BiVO_4_/Fe_2_TiO_5_ photoanodes; OCP and EIS curves; KPFM images of photoanodes; TAS curves for BiVO_4_ and BiVO_4_/Fe_2_TiO_5_ photoanodes; fast-scan CV scans; Mott–Schottky curves; and faradaic efficiency and stability (PDF)
