# Supplementary material for: Enhancing Photoelectrochemical Water Oxidation Using Ferromagnetic Materials and Magnetic Fields
Source: J Am Chem Soc. 2024 Dec 4;146(50):34681–9. doi: 10.1021/jacs.4c13017 (PMC12312158; doi:10.1021/jacs.4c13017)
Supplement: Supplementary file 1 [file ja4c13017_si_001.pdf]

Supporting Information

Enhancing photoelectrochemical water oxidation using ferromagnetic materials and magnetic field

Qingjie Wang[a,b], Louise I. Oldham[c], Alfredo Giner-Requena[b], Zeyuan Wang[a], Daniele Benetti[c], Salvador Montilla-Verdú[b], Rong Chen[a], Dongfeng Du[a], Teresa Lana-Villarreal[b], Ulrich Aschauer[d], Néstor Guijarro[b]*, James Robert Durrant[c]*, Jingshan Luo[a,e,f,]*

[a]*Institute of Photoelectronic Thin Film Devices and Technology, State Key Laboratory of Photovoltaic Materials and Cells, Tianjin Key Laboratory of Efficient Solar Energy Utilization, Ministry of Education Engineering Research Center of Thin Film Photoelectronic Technology, Nankai University, Tianjin 300350, China*

[b]*Institute of Electrochemistry, Universidad de Alicante, Apartat 99, E-03080 Alacant, Spain*

[c]*Centre for Processable Electronics, Department of Chemistry, Imperial College London, London, SW7 2AZ, United Kingdom*

[d]*Department of Chemistry and Physics of Materials, University of Salzburg, Jakob-Haringer-Str. 2A, Salzburg 5020, Austria*

[e]*Frontiers Science Center for New Organic Matter, Nankai University, Tianjin 300071, China*

[f]*Haihe Laboratory of Sustainable Chemical Transformations, Tianjin 300192, China*

**Email: jingshan.luo@nankai.edu.cn, j.durrant@imperial.ac.uk, nestor.guijarro@ua.es*

Experimental Section

Synthesis of the $BiVO_4$ photoanodes: The nanoporous $BiVO_4$ photoanode was prepared using a previously reported method with slight modifications[S1]. Typically, 3.32 g KI was dissolved in 50 mL of deionized water and adjusted the pH to 1.6~1.8 with HNO_3. Then, 0.974 g $Bi(NO_3)_3{\cdot}5H_2O$ was added to the solution until it turned into an orange-red transparent solution. Subsequently, 20 mL ethanol solution containing 0.497 g p-benzoquinone was mixed with the previous solution and stirred vigorously for 30 min, the supernatant was then filtered through a 0.22 μm filtration membrane to obtain the electrodeposition solution. The optimal cathodic deposition of BiOI (1×1 cm^2) was performed at -0.1 V vs Ag/AgCl for 8 min at room temperature using a CHI 760e electrochemical workstation, FTO served as the working electrode (WE), Pt foil as the counter electrode (CE), and Ag/AgCl (4 M KCl) as the reference electrode (RE), respectively. Following this, the BiOI film was directly converted into the $BiVO_4$ photoanode. Dimethyl sulfoxide (DMSO) solution containing 0.2 M vanadyl acetylacetonate ($VO(acac)_2$) was coated on the BiOI electrode and annealed in air at 450 °C for 2 h with a ramping rate of 2°C/min to convert BiOI into $BiVO_4$. Finally, the excess V_2O_5 was removed by soaking the film in 1 M NaOH solution for 20 min with gentle stirring, and the film was rinsed with deionized water and dried in air to obtain the pure $BiVO_4$ photoanode.

Synthesis of the $BiVO_4/Fe_2TiO_5$: The $BiVO_4/Fe_2TiO_5$ photoanode was prepared by a hydrothermal method. Initially, 5 mM titanium isopropoxide ($C_{12}H_{28}O_4Ti$) was dissolved in absolute ethanol and stirred vigorously to prevent hydrolysis of Ti. Subsequently, 10 mM iron acetylacetonate ($Fe(acac)_3$) was dispersed into the above solution until the color of the solution turned into clear orange-red. The as-prepared $BiVO_4$ photoanode was transferred into an autoclave and the hydrothermal reaction was conducted at 120°C for 3 h, 6 h, 9 h, and 12 h, respectively. The obtained photoanode was rinsed with deionized water and dried at room temperature. Lastly, the sample was calcined at 600°C for 2 h (ramping rate=5°C/min) in the air to obtain the $BiVO_4/Fe_2TiO_5$ photoanode. The optimum hydrothermal reaction time was 9 h.

Synthesis of the Fe_2TiO_5 powder and Fe_2TiO_5 photoanode: the Fe_2TiO_5 powder was synthesized by preparing the same ethanol solution containing $C_{12}H_{28}O_4Ti$ and $Fe(acac)_3$ that was used to prepare the $BiVO_4/Fe_2TiO_5$ photoanode. The solution was filtered and the obtained sample was washed with ethanol/water three times and dried at 80°C for 6 h. Lastly, the sample was calcinated at 600 °C to obtain the phase pure Fe_2TiO_5 powder. The Fe_2TiO_5 photoanode was fabricated by electrophoretic deposition methods. Typically, Fe_2TiO_5 powder (40 mg) was dispersed in acetone (50 mL) with iodine (10 mg). The addition of iodine gave H^+ by the reaction with acetone and thus made particles positively charged. Two FTO glasses were immersed in the solution in parallel at a distance of 2 cm and 10 V of voltage was applied between the electrodes for 1~2 h using a CHI-760e or Autolab electrochemical workstation. The coated area was about 2×2 cm^2 (denoted "Fe_2TiO_5" electrode). Furthermore, the electrode was heat treated in the air at 600°C for 2 h to better connection between particles and electrode.

Preparation of TiO_2 photoanode: the TiO_2 nanorod array was synthesized by the previously reported methods.[S2] Briefly, 0.175 mL of tetrabutyl titanate was dripped dropwise into the mixed solution of H_2O/HCl with equal volume (6 mL) of H_2O and HCl (36.5-38 wt%) under continuous stirring until the solution yurns completely transparent. The FTO and resulting solution were transferred into a Teflon-lined stainless-steel autoclave and heated to 150°C for 20 h. The obtained TiO_2 nanorods were washed with deionized water and annealed at 500 °C for 2 h (heating rate: 5 °C/min).

Preparation of WO_3 photoanode: the WO_3 nanoplate array was prepared via the previously reported hydrothermal method with slight modifications.[S3] Initially, 6 mL HCl (3 M), 0.04 g $Na_2WO_4 \cdot 2H_2O$, and 0.2 g $(NH_4)_2C_2O_4$ were added into 70 mL H_2O with constant stirring at room temperature until the solution became completely transparent. The resulting solution and pre-treated FTO were placed in the autoclave and the hydrothermal reaction was performed at 150°C for 2 h. Finally, the as-prepared WO_3 array was washed thoroughly with distilled water and annealed in air at 500 °C for 2 h (heating rate: 5 °C/min).

Preparation of Fe_2O_3 photoanode: the Fe_2O_3 nanorod array was synthesized via the

previously reported method.[S4] Typically, 0.15 M $FeCl_3 \cdot 6H_2O$ and 1 M $NaNO_3$ were dissolved into 20 mL H_2O with vigorous stirring for 30 min, then the pre-treated FTO and resulting solution were transferred into the autoclave and heated to 95 °C for 6 h. The as-obtained Fe_2O_3 precursor sample was washed with deionized water and calcined in air at 550 °C for 2 h (heating rate: 2 °C/min) and further heated to 650 °C for 20 min (heating rate: 10 °C/min) to improve the crystallinity of Fe_2O_3 photoanode.

Preparation of TiO_2/Fe_2TiO_5, WO_3/Fe_2TiO_5 and Fe_2O_3/Fe_2TiO_5 photoanode: the preparation method of TiO_2/Fe_2TiO_5, WO_3/Fe_2TiO_5 and Fe_2O_3/Fe_2TiO_5 photoanode is similar to that of $BiVO_4/Fe_2TiO_5$ except that $BiVO_4$ is replaced by TiO_2, WO_3 and Fe_2O_3.

Structural and spectroscopic characterizations.
The crystal structure of the as-prepared samples was investigated using X-ray diffraction (XRD) and measurements were performed on a Rigaku Ultima IV X-ray diffractometer (Rigaku, Japan) operating at 40 kV and 40 mA with the Cu Kα (λ = 0.154 18 nm) as the radiation source at a scanning rate of 10°/min and the scanning range was from 10 – 70°. The morphology of the as-prepared samples was imaged using a field-emission scanning electron microscope (FE-SEM, Apreo S 160 LoVac, FEI, and JSM-7800F, JEOL) and transmission electron microscope (TEM, JEM-2800, JEOL) equipped with an Energy dispersive spectrometry (EDS) analyzer. The HRTEM images were also obtained using the same transmission electron microscope. UV-visible diffuse reflectance spectra (DRS) of the photoanode were recorded on a Cary 5000 (Agilent) using $BaSO_4$ as the reference. X-ray photoelectron spectroscopy (XPS) was used to explore the surface elemental compositions and chemical states of the as-prepared photoanodes. XPS measurements were conducted on a ESCALAB 250 Xi XPS spectrometer (Thermo Fisher Scientific) equipped with a Al Kα monochromator X-ray source with a constant pass energy of 100 eV. The binding energy was calibrated using the C 1s (284.8 eV) as the reference for all the measurements and the data was fitted using XPS Peak41 software. The UPS spectra were performed on an Auger electron spectrometer (Thermo SCIENTIFIC ESCALAB 250 Xi) using a He I (21.22 eV) excitation line and the work function was calculated from the UPS spectra by subtracting the secondary electron cutoff edge. Note that the $BiVO_4$ and Fe_2TiO_5 thin films tested by UPS were not subjected to any PEC test. The magnetization of the samples, prior the UPS treatment was performed by placing the thin films onto the magnet's surface for 24 h. The magnetic properties were measured on a vibrating sample magnetometer (VSM, Quantum Squid-vsm). The conducting Kelvin probe spectroscopy was carried out on an atomic force microscope (AFM, NTEGRA Prima) in the dark and under illumination conditions to measure the distribution of surface photovoltage on the photoanodes. Magnetic Force Microscope (MFM) tips used were MESP-V2 from Briker with 400 Oe coercivity, optimized for non-perturbative magnetic imaging with high spatial resolution (Bruker, Dimension Icon).

Transient absorption spectroscopy (TAS) measurements were performed using a custom-built system with a Nd:YAG 355 nm laser (Ultra Quantel Big Sky Laser, Litron

Stable Resonator Nano S 120-20) excitation pulse set to a power output of ~300 μJ cm^{-2} (± 7%) and a pulse frequency of 1 Hz. Laser pulses were detected by a silicon detector (ThorLabs DET10A/M), which generated a voltage signal to trigger data recording on a digital phosphor oscilloscope (Tektronix DPO 3012) and a DAQ card (National Instruments NI USB-6251). The probe light source was a tungsten halogen lamp (Osram 64640HLX, Bentham IL1 lamp housing, Bentham 610 power supply)., and was focused and collimated through a series of lenses. Long pass filters minimized sample excitation from the probe lamp. A color wheel (ThorLabs FW102C) and monochromator (Oriel Cornerstone 130 1/8 m monochromator) selected for the specific wavelength and the transmitted light was collected by a detector (Costronics Optical Transient Pre-Amplifier - Mk2). The detected signal was processed through an optical transient amplifier (Costronics 2008 Optical Transient Amplifier) and recorded by the oscilloscope (μs-ms timescale data) and the DAQ card (ms-s timescale data). Data for each TAS decay were averaged over 40-250 laser pulses. A B trace was recorded with the shutter closed and subtracted from the A trace. The system was managed by custom Labview software, and the data were smoothed using a custom Matlab application. The sample served as the working electrode in a three-electrode cell setup, with a platinum mesh counter electrode, a Ag/AgCl (sat. KCl) reference electrode, and 1 M KBi electrolyte (pH 9.5). Electrical bias was applied using an Autolab potentiostat (PGSTAT101) and controlled using Nova software. For measurements to investigate the magnetic field effect, a Nd magnet was clamped behind the three-electrode cell (closest to the Fe_2TiO_5 coating side). For measurements where the magnet remained in place during TAS measurements, two stacked magnets with a countersunk hole were used to allow light to pass through the hole in the magnet to reach the detector. For measurements where the magnet was removed before further TAS measurements, a stronger square magnet was used.

Photoelectrochemical measurements

The photoelectrochemical water splitting performance of prepared photoanodes was studied using a CHI 760E electrochemical workstation (CH Instruments Co.,Shanghai, China) and a standard three-electrode cell with all quartz windows to allow UV-light to pass through. A class AAB solar simulator (HAL-320) was adopted as a light source, and the light intensity was carefully calibrated to 100 mW/cm^2 using standard silicon diodes. A 1 M KBi buffer (pH=9.5) with or without Na_2SO_3 was used as the electrolyte for all the PEC tests. The exposed area of the photoanodes was 1 cm^2. The linear sweep voltammetry (LSV) curves were recorded using a scan range from -0.8 V to 0.6 V vs. Ag/AgCl in the anodic direction with a scan rate of 10 mV/s. The chopped LSV curve measurements were performed using a switching on/off time of 2 s without other changes to the test conditions. For TiO_2, TiO_2/Fe_2TiO_5, WO_3 and WO_3/Fe_2TiO_5 photoanode, 0.2 M Na_2SO_4 (pH=6.8) was selected as the electrolyte, but for Fe_2O_3 and Fe_2O_3/Fe_2TiO_5, 1 M NaOH (pH=13.6) was chosen as electrolyte. The stability of the $BiVO_4/Fe_2TiO_5$ photoanode was measured under AM 1.5 G illumination for 6 h under an applied potential of 1.23 V vs RHE with and without an external magnetic field. Generally speaking, and unless otherwise specified in the figure caption, the same

sample was initially tested in the absence of the magnetic, and subsequently, under the influence of the external magnetic field by placing the non-reflective magnet behind the photoanode. And for other tests, fresh samples were used for the test in the absence and in the presence of the magnetic field.

All measured potentials were converted to reversible hydrogen electrode (vs RHE) potentials using the Nernst equation:

$E_{RHE}=E_{Ag/AgCl}+0.0592pH+E^0_{Ag/AgCl}$

where $E_{Ag/AgCl}$ is the measured potential and $E^0_{Ag/AgCl}$ = 0.197 V (vs Ag/AgCl) at 25 °C.

Mott-Schottky (M-S) curve was also measured under dark conditions with the applied potential of -0.8~-0.1 V vs Ag/AgCl, the applied frequency was 1000 Hz and the amplitude was 10 mV/s. Furthermore, the carrier density (N_d) and depletion width were obtained from M-S curves by the following formula:

$$N_d=(2/e_0\varepsilon\varepsilon_0)[d(1/C^2)/dV]^{-1}$$

The electron charge (e_0) is 1.6×10^{-19} C, ε is relative permittivity (ε=68) for $BiVO_4$,[S5] ε_0 is vacuum permittivity (8.86×10^{-12} F/m), C is the space charge capacitance in the semiconductor, and V is the applied bias at the electrode, $d(1/C^2)/dV$ was the straight slope, N_d is the charge carrier density.

The incident photon to current efficiency (IPCE) measurements were carried out using the monochromatic light generated from a 300 W high uniformity integrated solar simulator (HAL-320, Asahi Spectra, Japan) coupled with a monochromator (Zolix, Omni-λ 300). A potential of 1.23 V vs RHE was applied and KBi was used as the electrolyte, the wavelength range was from 300 – 550 nm with a 10 nm interval. The IPCE result was calculated using the following equation:

$$IPCE\ (\%) = \frac{1240\ \times\ J\ (\mathrm{mA/cm^2})}{\lambda\ (\mathrm{nm}) \times\ P\ (\mathrm{mW/cm^2})} \times 100$$

Where J is the measured photocurrent density at each specific wavelength, λ is the wavelength of the incident light and P is the light power density at a specific wavelength.

The half-cell solar-to-hydrogen conversion efficiency (HC-STH) was calculated from the LSV curves under AM 1.5G illumination using the following equation:

$$\mathrm{HC-STH} = \frac{J \times (1.23 - V_{bias})}{P_{light}} \times 100\%$$

Where J is the photocurrent density, V_{bias} is the applied potential vs RHE, P_{light} is the illumination power density (100 mW/cm^2) of the simulated sunlight.

The photoelectrochemical impedance spectroscopy (PEIS) and particularly the Nyquist plots were recorded at 0.6 V vs RHE under illumination with a small AC

amplitude of 10 mV in the frequency range of 0.01 – 100 kHz with and without an external magnetic field, the measured data was fitted with Zview software.

The light-harvesting efficiency (LHE, η_{LH}) was calculated from the UV-Vis DRS spectra and the following the equation:

$$\eta_{LH} = 1 - 10^{-A(\lambda)}$$

The absorbed photon-to-current efficiency (APCE) and the light harvesting efficiency (LHE) were calculated based on the following formula:

$$APCE = \frac{IPCE}{\eta_{LH}}$$

The charge transfer efficiency (η_{trans}) and separation efficiency (η_{sep}) were obtained by using the following equations:

$$\eta_{trans} = \frac{J_{water}}{J_{sulfite}}$$

$$\eta_{sep} = \frac{J_{sulfite}}{J_{abs}}$$

Where the maximum possible photocurrent (J_{abs}) was calculated from the standard solar spectrum and the light-harvesting efficiency using the following equations:

$$J_{abs} = \int_{\lambda 1}^{\lambda 2} \mathrm{N}(\lambda)AM1.5 \ \mathrm{LHE}\ (\lambda)\ \mathrm{d}\lambda$$

where $\mathrm{N}(\lambda)AM1.5 = \frac{\Phi(\lambda)\mathrm{AM1.5}}{\mathrm{E}} = \frac{\lambda\ \Phi(\lambda)\mathrm{AM1.5}}{\mathrm{h\ c}}$

$$LHE = 1\text{-}10^{-\mathrm{A}(\lambda)}$$

$$J_{abs} = \frac{\mathrm{q}}{\mathrm{h\ c}} \int_{300\ \mathrm{nm}}^{515\ \mathrm{nm}} \lambda\ \Phi(\lambda)\mathrm{AM1.5} \times (\ 1 - 10^{-\mathrm{A}(\lambda)})\ \mathrm{d}\lambda$$

Here $N(\lambda)AM1.5$ (in units of $m^{-2}\ s^{-1}$) is the photon flux density under standard conditions (AM1.5G 100 mW cm^{-2}), $\Phi(\lambda)AM1.5$ (unit of $Wm^{-2}\ nm^{-1}$) is the energy of the photons generated by the solar simulator, E is the energy of one photon, λ (unit of nm) is the wavelength of the photon, $\hbar$ (6.626×10^{-34} J·s) is Planck's constant, q (1.602×10^{-19} C) is the charge of one electron, LHE represents the light-harvesting efficiency, $A(\lambda)$ is the measured absorbance of the photoelectrode.

The open-circuit potential (OCP) measurements of the prepared photoanodes were performed using 1 M KBi electrolyte (pH=9.5) under dark and illumination conditions.

Fast-Scan Cyclic voltammetry and estimate $Q_{r\text{-}ss}$: To analyze the holes trapped at the interface under illumination, the photoanode was kept at 2.0 V vs RHE under AM 1.5G illumination for 60 s to oxidize the surface, following by fast cyclic voltammetry starting from the higher applied potentials (from 2.0 V to 0.6 V vs RHE) at 0.3 V·s^{-1} for

4 consecutive cycles. The integration of the cathodic band centered at ca. 1.6 V vs RHE results in the estimated $Q_{r\text{-}ss}$.

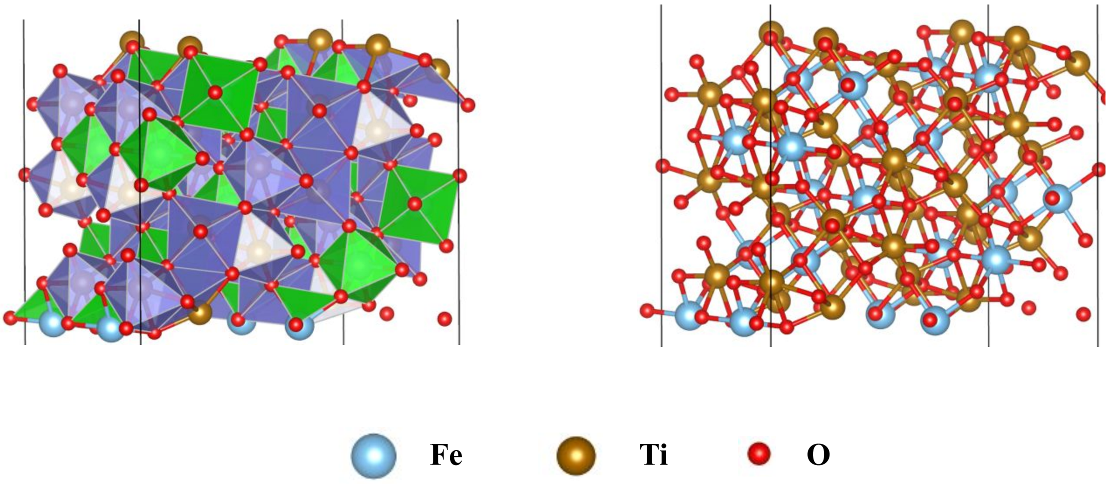

Figure S1. The crystal structure of Fe_2TiO_5.

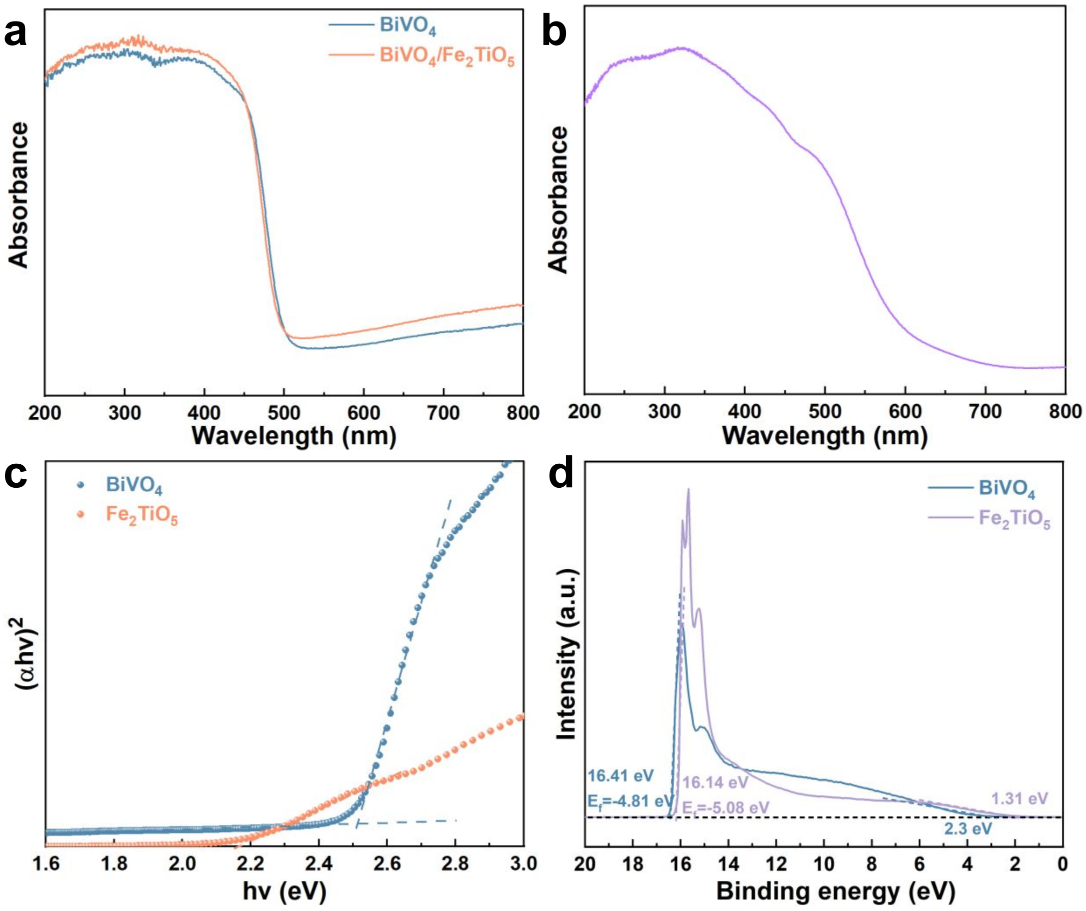

Figure S2. The absorption spectra of **a** $BiVO_4$ and $BiVO_4/Fe_2TiO_5$. **b** Fe_2TiO_5. **c** Tauc plots showing the band gaps of the $BiVO_4$ and Fe_2TiO_5 samples. **d** UPS spectra of $BiVO_4$ and Fe_2TiO_5 samples.

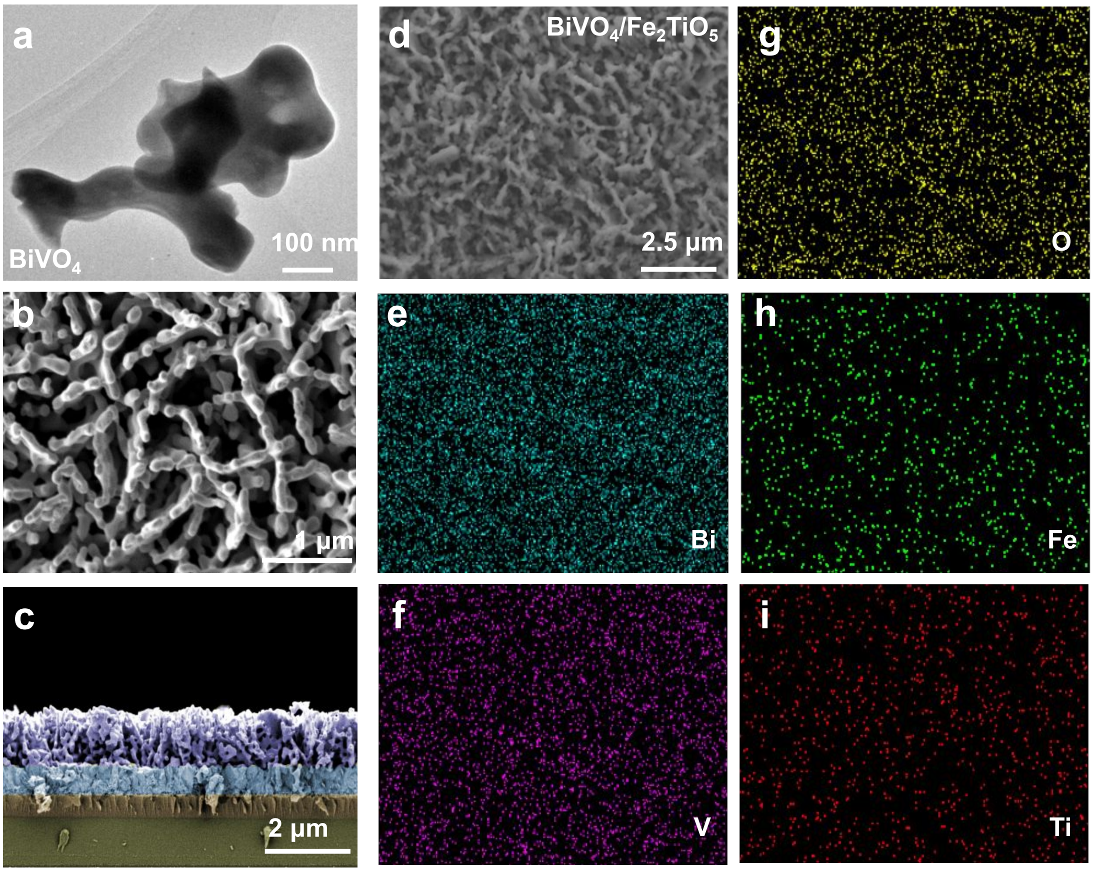

Figure S3. **a** TEM image, **b** Top-view SEM image and **c** Cross-section SEM image of the $BiVO_4$ photoanode. **d** Top-view SEM image of the $BiVO_4/Fe_2TiO_5$ photoanode. **e-i** corresponding STEM-EDX elemental mapping images for the $BiVO_4/Fe_2TiO_5$ anode showing the distribution of Bi, V, O, Fe and Ti.

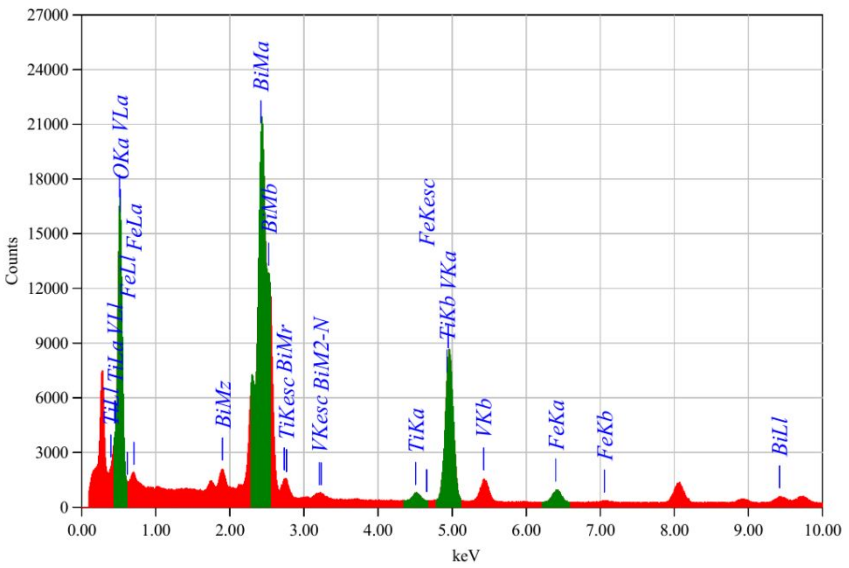

Thin Film Standardless Standardless Quantitative Analysis
Fitting Coefficient : 0.2851

Element	(keV)	Mass%	Counts	Sigma	Atom%	Compound	Mass%	Cation	K
O K	0.525	11.15	73713.53	0.06	52.50				0.3841
Ti K	4.508	0.54	4919.53	0.02	0.85				0.2795
V K	4.949	12.20	105037.63	0.08	18.05				0.2950
Fe K	6.398	1.17	8887.83	0.03	1.58				0.3353
Bi M (Ref.)	2.419	74.93	190293.92	0.31	27.02				1.0000
Total		100.00			100.00				

Figure S4. Energy Dispersive X-ray Spectroscopy (EDX) spectrum of the $BiVO_4/Fe_2TiO_5$ photoanode and the corresponding quantitative elemental analysis.

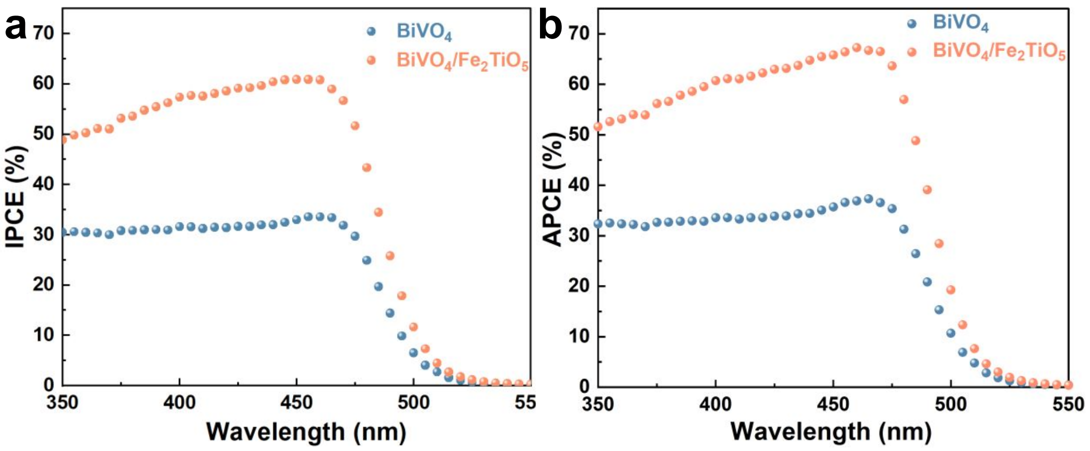

Figure S5. **a** IPCE and **b** APCE curves at 1.23 V vs RHE of $BiVO_4$ and $BiVO_4/Fe_2TiO_5$ photoanodes in 1 M KBi solution (pH 9.5).

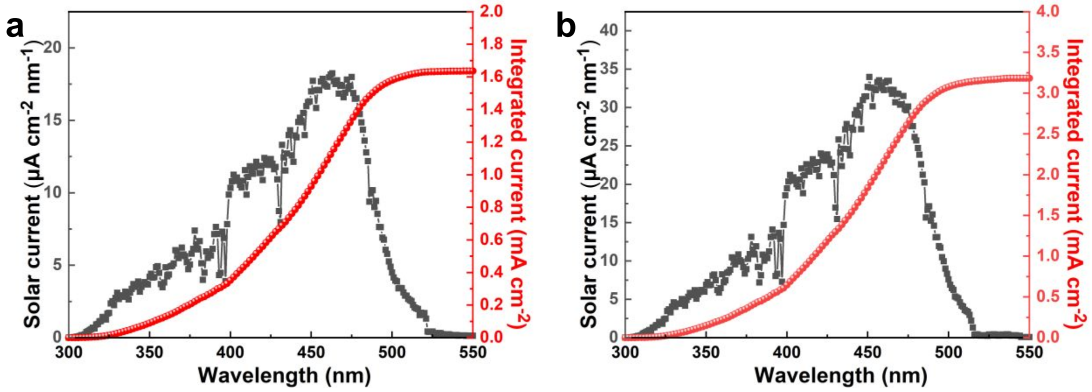

Figure S6. Solar photocurrent of **a** $BiVO_4$, and **b** $BiVO_4/Fe_2TiO_5$ photoanodes (left ordinate), and the integrated photocurrent density were derived from the IPCE measurement at 1.23 V vs RHE in 1 M KBi solution (right ordinate).

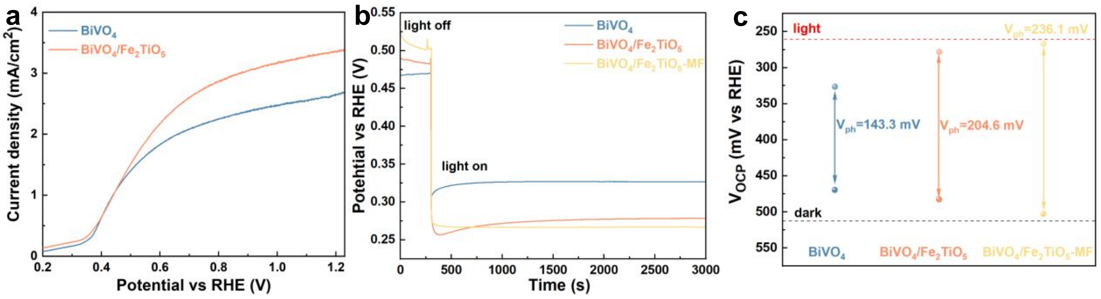

Figure S7. **a** J-V curves of the $BiVO_4$ and $BiVO_4/Fe_2TiO_5$ photoanodes measured under AM 1.5G irradiation with 0.2 M Na_2SO_3 in 1 M KBi (pH 9.5) as the hole scavenger. **b** The OCP difference values were measured under AM 1.5G illumination and dark conditions 1 M KBi (pH 9.5). **c** OCP diagrams of $BiVO_4$ and $BiVO_4/Fe_2TiO_5$ photoanode.

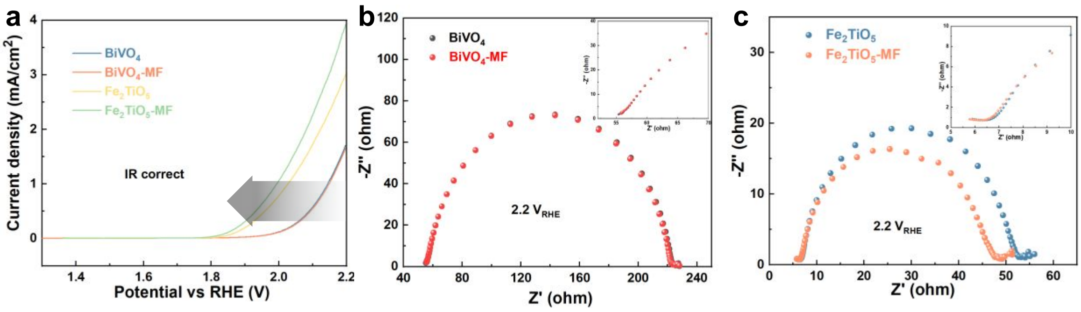

Figure S8. **a** the IR corrected LSV curves of $BiVO_4$ and Fe_2TiO_5 with or without applying MF in the 1 M KBi (pH 9.5) buffer solution. **b** electrochemical impedance spectroscopy (EIS) spectra of the $BiVO_4$ and $FeTiO_5$ film measured at 2.2 V vs RHE with and without MF.

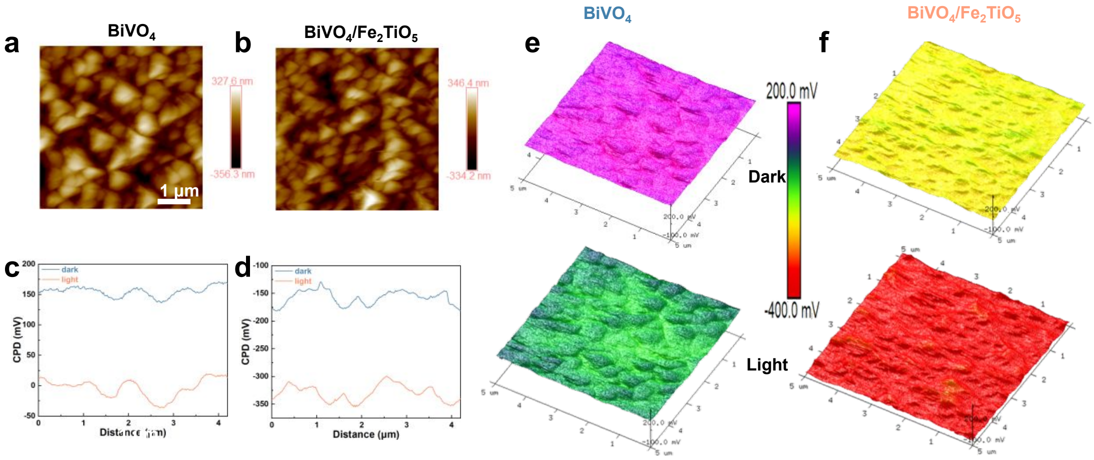

Figure S9. AFM topgraphical images of **a** $BiVO_4$ and **b** $BiVO_4/Fe_2TiO_5$ photoanodes. The average contact potential of **c** $BiVO_4$ and **d** $BiVO_4/Fe_2TiO_5$ under dark and light environments. KPFM images of **e** $BiVO_4$ and **f** Fe_2TiO_5 under dark and illumination conditions.

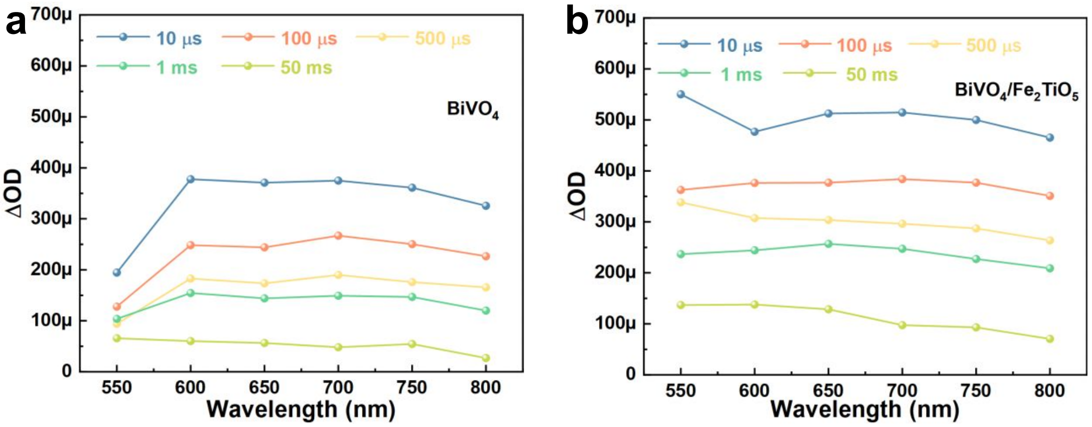

Figure S10. Transient absorption spectra of **a** $BiVO_4$ and **b** $BiVO_4/Fe_2TiO_5$ under an applied bias of 1.2 V_{RHE}. Data plotted at different time points after front-side laser excitation at 355 nm (~300 μJ cm-2, 1 Hz). Measurements were carried out in 1 M KBi buffer solution, pH 9.5.

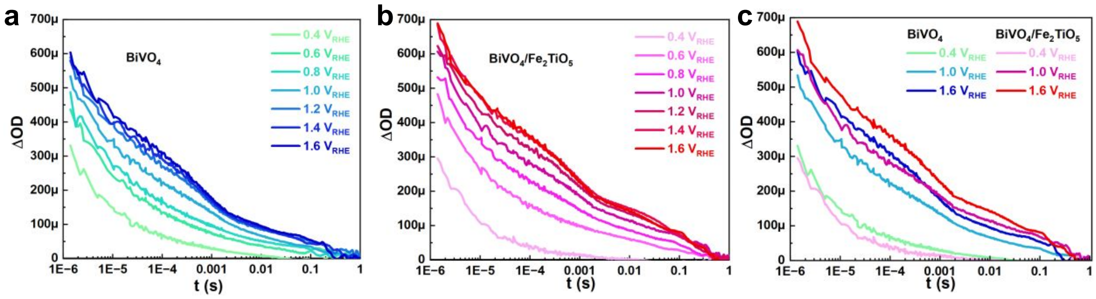

Figure S11. Transient absorption decays of **a** $BiVO_4$ and **b** $BiVO_4/Fe_2TiO_5$ under a range of anodic applied potentials from +0.4 V_{RHE} to +1.6 V_{RHE}. **c** Compares the decays of $BiVO_4$ and $BiVO_4/Fe_2TiO_5$ at three of these potentials. Decay traces probed at 700 nm after front-side laser excitation at 355 nm (~300 μJ cm^{-2}, 1 Hz). Measurements carried out in 1 M KBi buffer, pH 9.5.

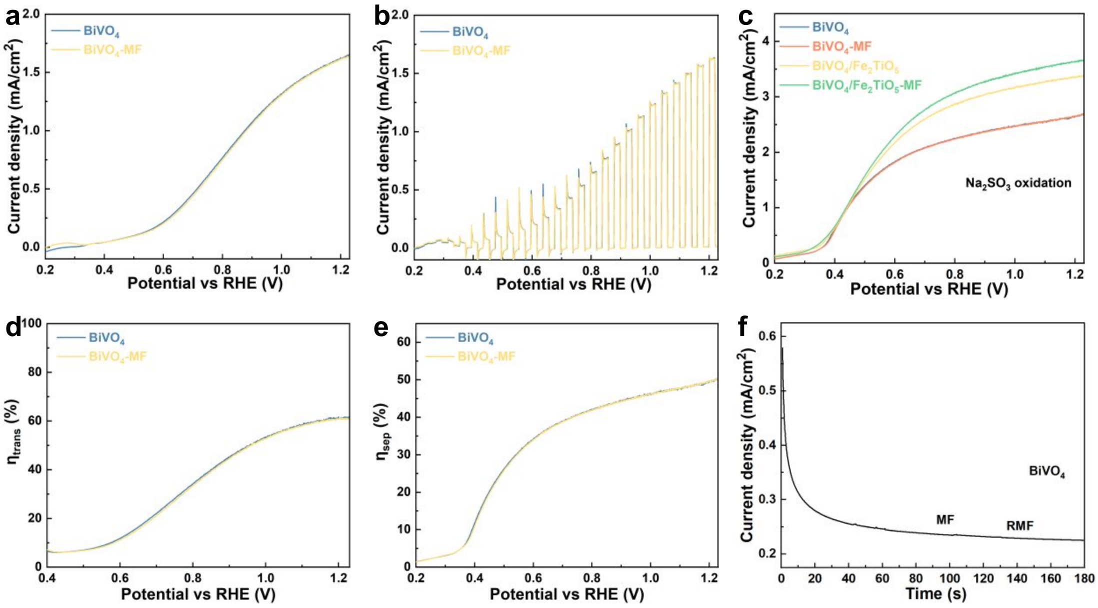

Figure S12. **a** *J*-*V* curves, **b** chopped *J*-*V* curves of $BiVO_4$ photoanode with and without MF, **c** J-V curves of $BiVO_4$ and $BiVO_4/Fe_2TiO_5$ photoanode with and without MF in the 1 M KBi solution including the 0.2 M Na_2SO_3 as hole scavenger. **d** charge transfer efficiency (η_{trans}) and **e** charge separation efficiency (η_{sep}), of $BiVO_4$ photoanode with and without MF. **f** transient magnetic respond photocurrent of $BiVO_4$ photoanode at 1.23 V vs RHE after application of MF and removal of MF.

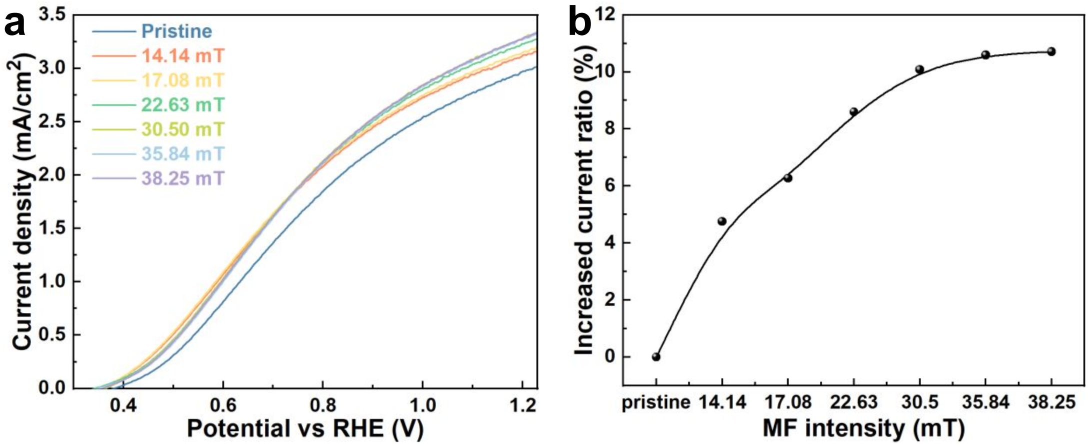

Figure S13. **a** *J-V* curves of $BiVO_4/Fe_2TiO_5$ photoanode with different magnetic field intensity under back illumination in KBi solution. **b** The increased photocurrent ratio of $BiVO_4/Fe_2TiO_5$ in the different magnetic field intensity.

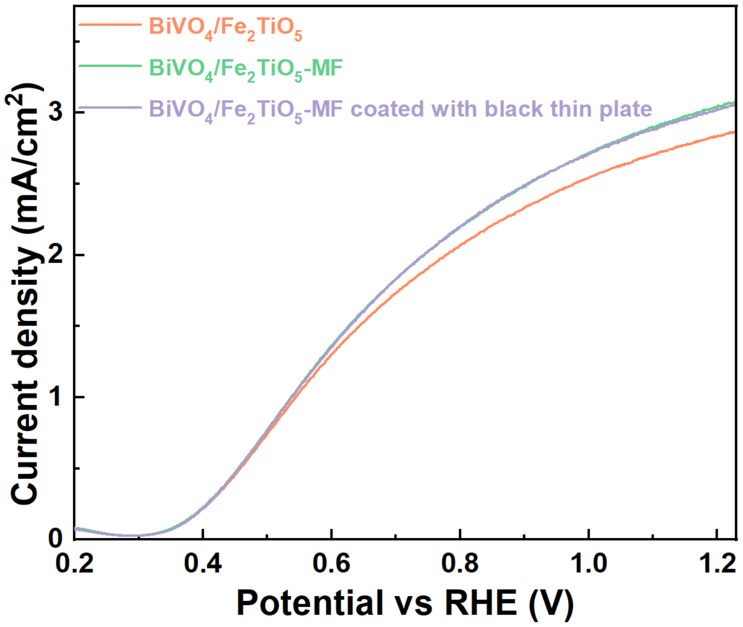

Figure S14. *J*-*V* curves obtained for the pristine $BiVO_4/Fe_2TiO_5$ photoanode in the absence of the magnet, and in the presence of the magnet without and with a black coating to eliminate the reflection of incident light.

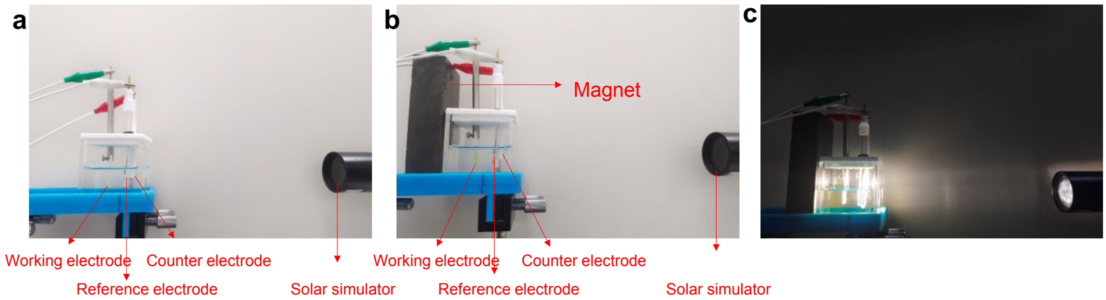

Figure S15. Pictures of the PEC setup without (a) including the magnet (b) and the PEC experiment under AM 1.5 G illumination with the magnet (c).

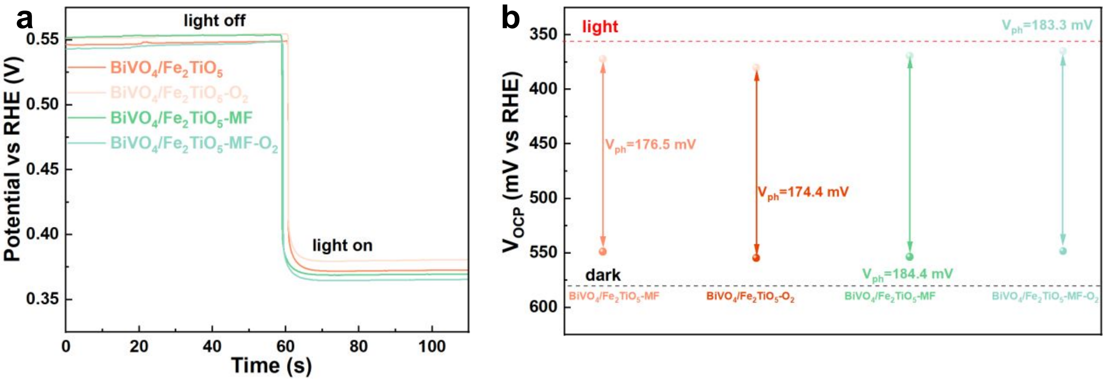

Figure S16. **a** The OCP difference values were measured under AM 1.5G illumination and dark conditions 1 M KBi (pH 9.5) with or without O_2 purging. **b** OCP diagrams of $BiVO_4$ and $BiVO_4/Fe_2TiO_5$ photoanode.

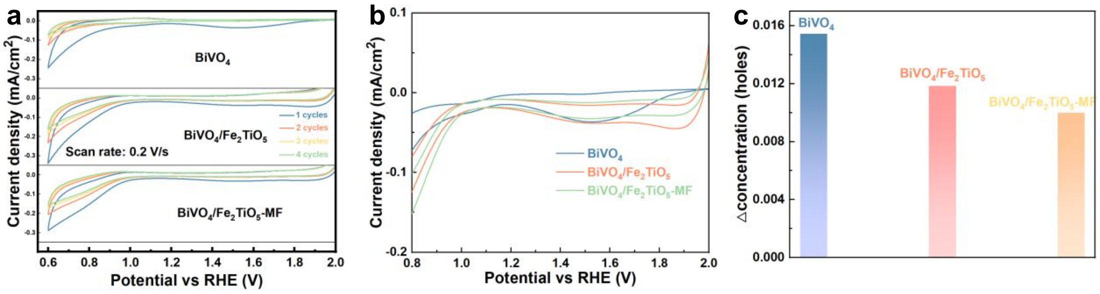

Figure S17. **a, b** FS-CVs recorded in the dark at 0.3 V/s for four cycles immediately after holding the potential at 2 V_{RHE} for 60 s under illumination of $BiVO_4$, $BiVO_4/Fe_2TiO_5$ and $BiVO_4/Fe_2TiO_5$-MF photoanodes. **c** the accumulated charge of $BiVO_4$, $BiVO_4/Fe_2TiO_5$ and $BiVO_4/Fe_2TiO_5$-MF photoanodes.

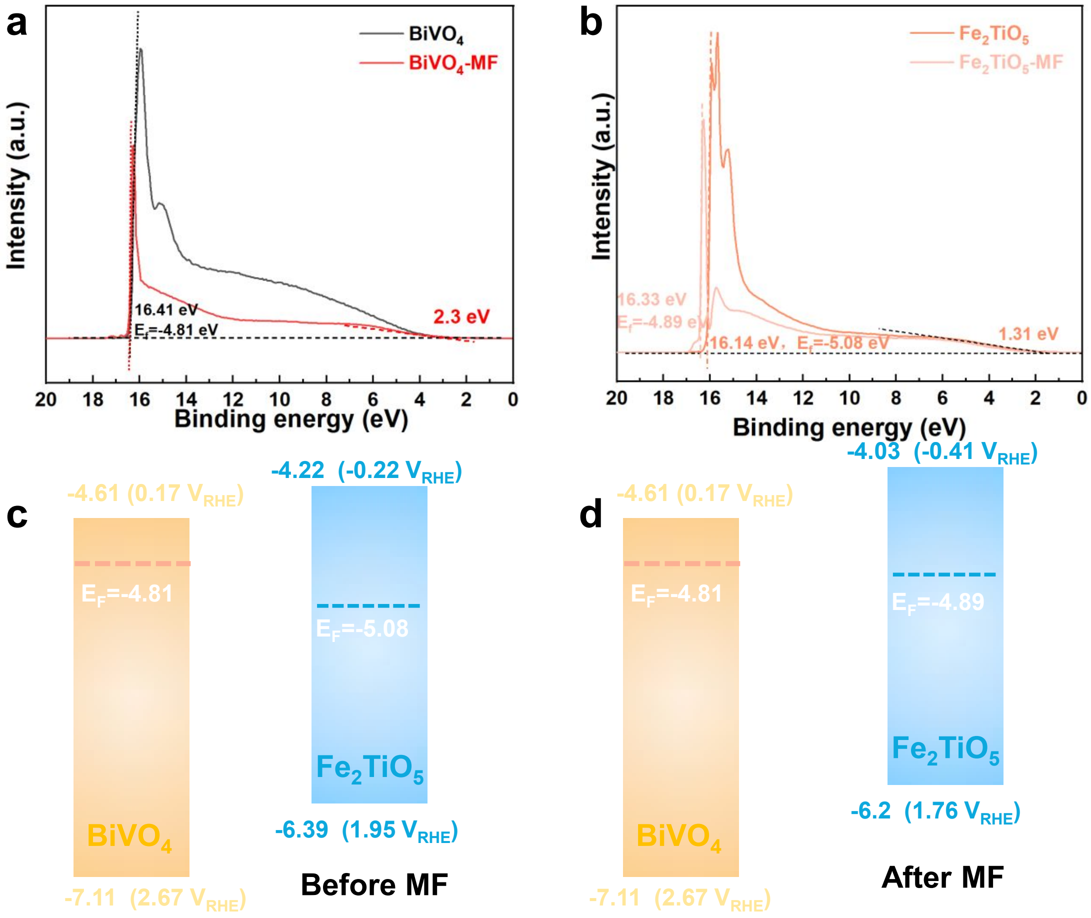

Figure S18. UPS spectra of **a** $BiVO_4$ and **b** Fe_2TiO_5 with and without MF. **c**, **d** the calculated energy band structure of $BiVO_4$ and Fe_2TiO_5 with and without external MF.

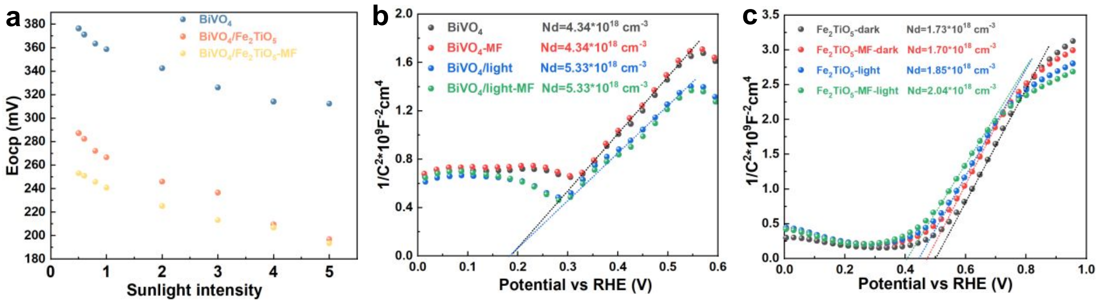

Figure S19. **a** the OCP values of $BiVO_4$, $BiVO_4/Fe_2TiO_5$ and $BiVO_4/Fe_2TiO_5$-MF at different solar illumination intensities. **b**, **c** Mott-Schottky plots of $BiVO_4$ and $BiVO_4/Fe_2TiO_5$ films under dark and illumination with and without MF.

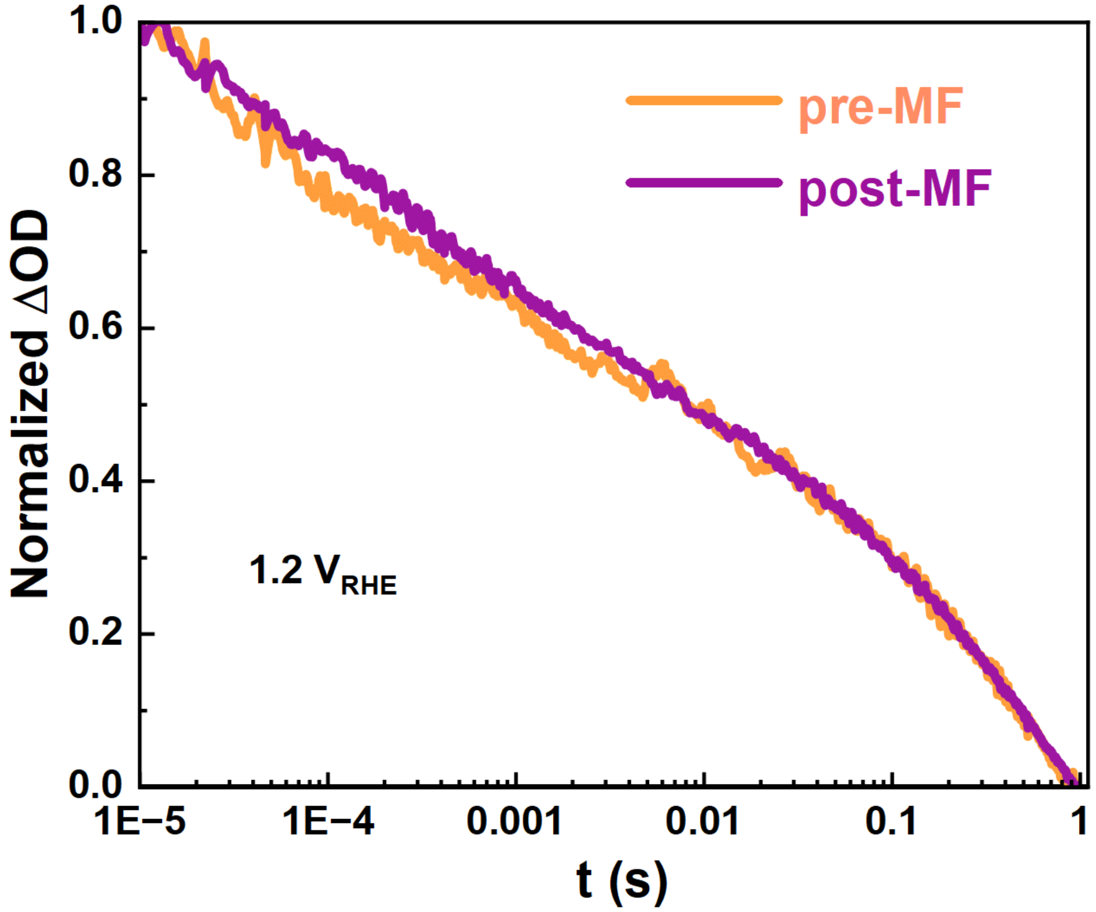

Figure S20. Transient absorption decays of $BiVO_4/Fe_2TiO_5$ under an applied bias of 1.2 V_{RHE} , before (orange) and after (purple) exposure to an external magnetic field, normalized at 10 μs. For the magnetisation process, the magnet was placed in front of the sample for 1.5 h and then removed before obtaining the second TAS trace. Decay traces probed at 650 nm after back-side laser excitation at 355 nm (~300 μJ cm^{-2}, 1 Hz). Measurements carried out in 1 M KBi buffer, pH 9.5. MF = magnetic field.

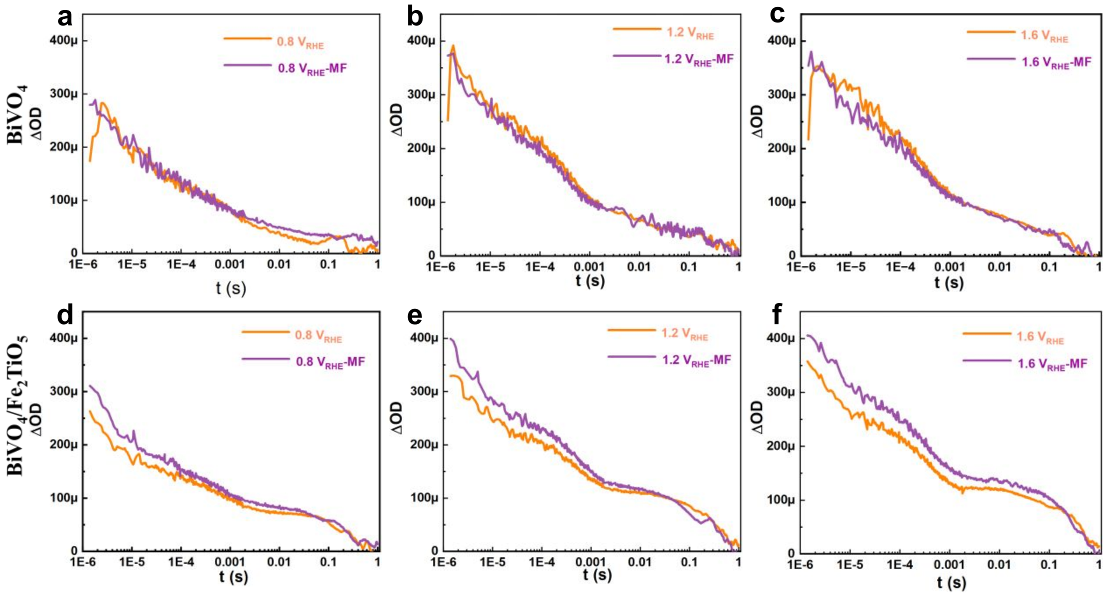

Figure S21. Transient absorption decays of $BiVO_4$ (**a-c**) and $BiVO_4/Fe_2TiO_5$ (**d-f**) under an applied bias of 0.8 V_{RHE} (**a**, **d**), 1.2 V_{RHE} (**b**, **e**) and 1.2 V_{RHE} (**c**, **f**) with (purple) and without (orange) an external magnetic field (MF). For the magnetisation process, the magnet was placed in front of the sample and left in place while obtaining the second TAS trace. Decay traces probed at 650 nm after back-side laser excitation at 355 nm (~300 μJ cm^{-2}, 1 Hz). Measurements carried out in 1 M KBi buffer, pH 9.5.

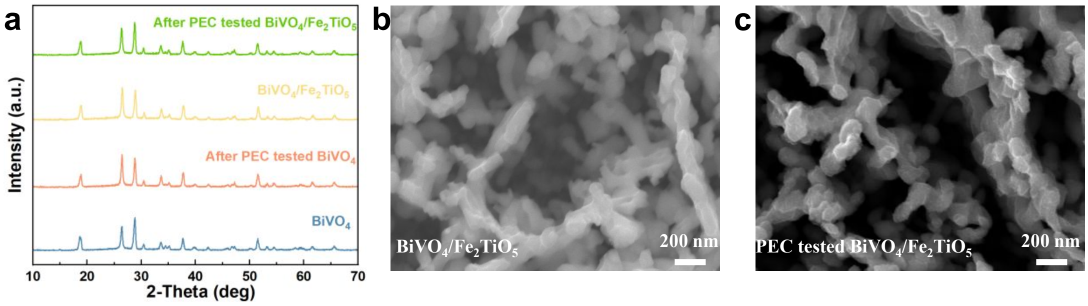

Figure S22. **a** XRD spectra of $BiVO_4/Fe_2TiO_5$ photoanodes before and after PEC testing. **b** and **c** SEM images of the $BiVO_4/Fe_2TiO_5$ photoanodes before and after PEC testing in 1M KBi (pH 9.5) solution for 2 h.

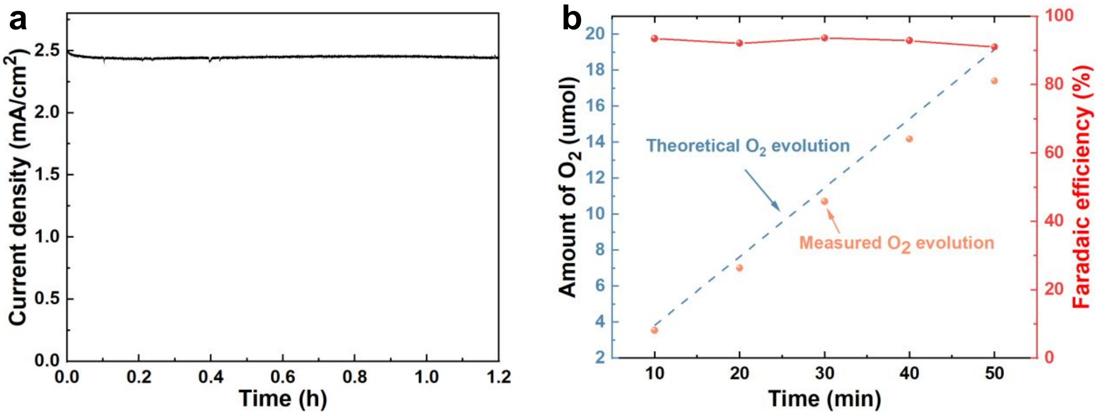

Figure S23. **a** the stability test of $BiVO_4/Fe_2TiO_5$ photoanode recorded at 0.8 V vs RHE under AM 1.5G illumination for 1.2 h, this current was also used to obtain the Faradaic efficiency. **b** the amount of oxygen produced by the $BiVO_4/Fe_2TiO_5$ photoanode and the calculated Faradaic efficiency. (Dotted lines and scattered points indicate the theoretical and measured values, respectively).

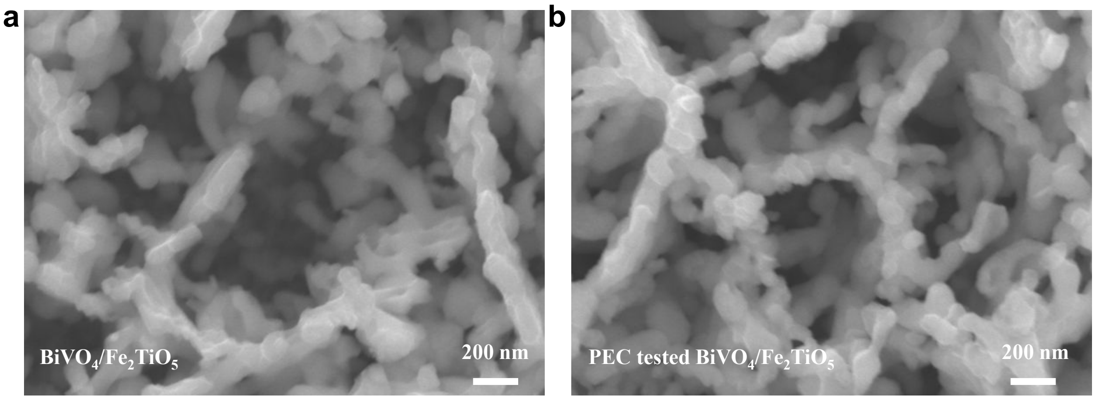

Figure S24. **a** SEM images of the freshly prepared $BiVO_4/Fe_2TiO_5$ photoanode and **b** the $BiVO_4/Fe_2TiO_5$ photoanode after PEC testing in 1M KBi (pH 9.5) solution for 2 h with external magnetic field.

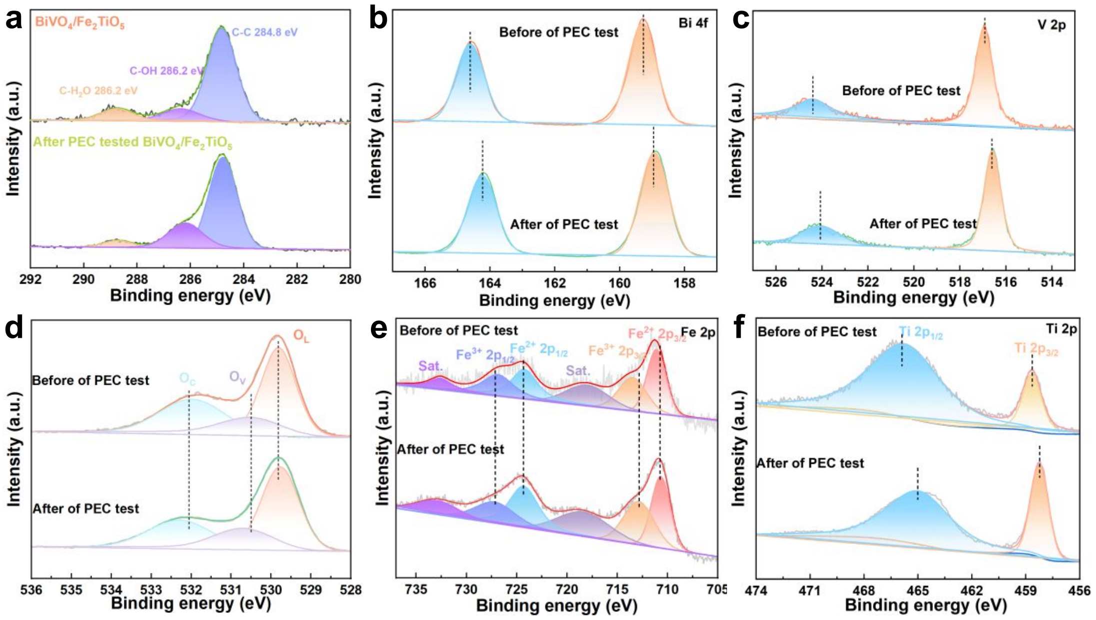

Figure S25. The high-resolution XPS spectra of **a** C 1s, **b** Bi 4f, **c** V 2p, **d** O 1s, **e** Fe 2p and **f** Ti 2p for $BiVO_4/Fe_2TiO_5$ before and after PEC testing in 1 M KBi (pH 9.5) with external magnetic field for 6 h.

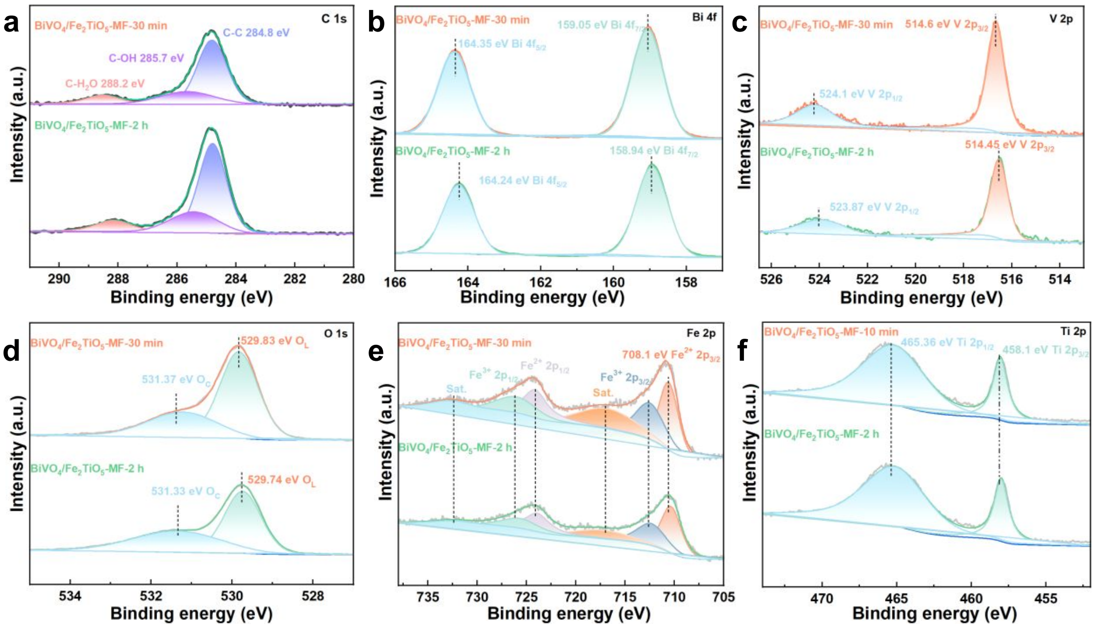

Figure S26. The high-resolution XPS spectra of **a** C 1s, **b** Bi 4f, **c** V 2p, **d** O 1s, **e** Fe 2p and **f** Ti 2p of $BiVO_4/Fe_2TiO_5$ after PEC testing in 1 M KBi (pH 9.5) for 30 min and 2 h with external magnetic field.

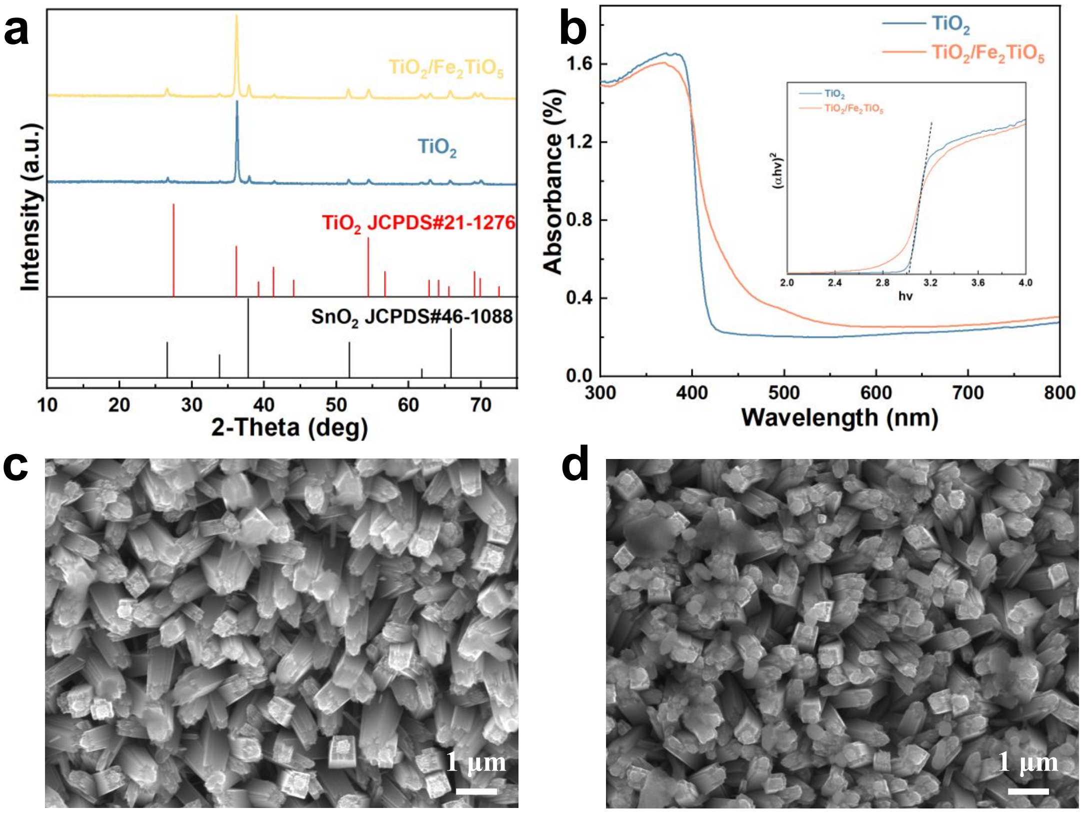

Figure S27. **a** XRD patterns and **b** UV-vis absorption spectra of TiO_2 and TiO_2/Fe_2TiO_5 photoanode. SEM imanges of **c** pristine TiO_2 and **d** TiO_2/Fe_2TiO_5 photoanode.

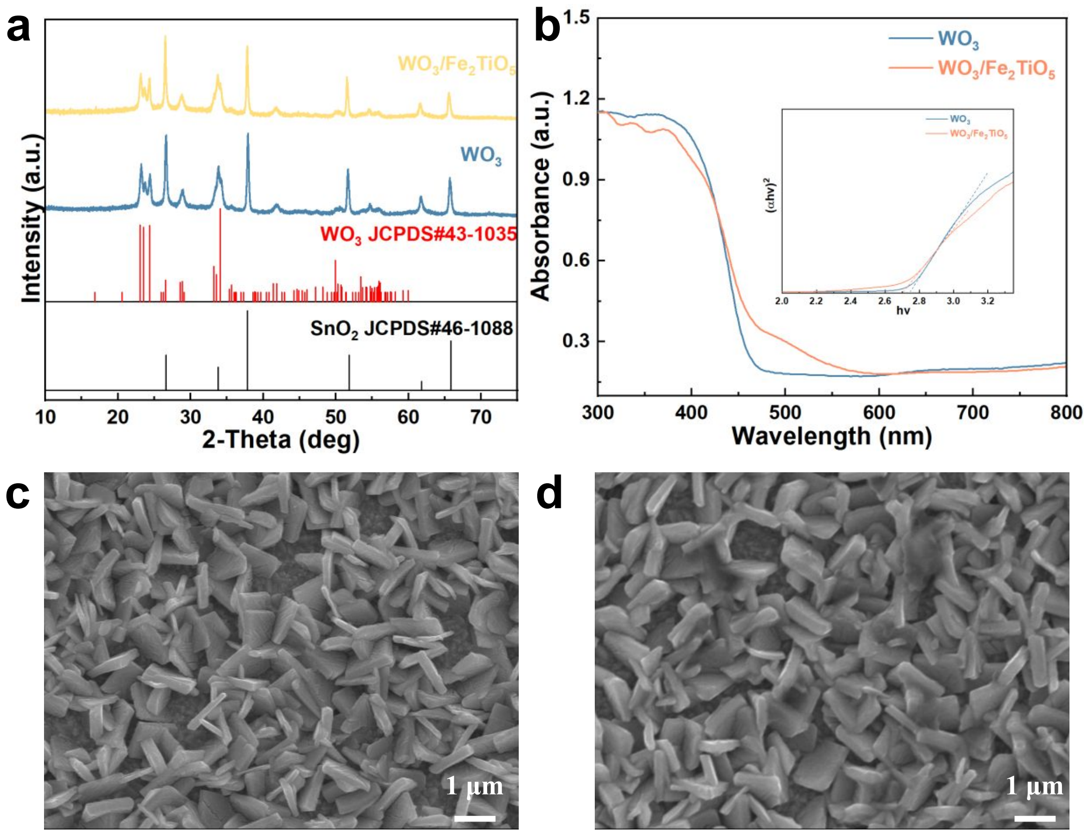

Figure S28. **a** XRD patterns and **b** UV-vis absorption spectra of WO_3 and WO_3/Fe_2TiO_5 photoanode. SEM imanges of **c** pristine WO_3 and **d** WO_3/Fe_2TiO_5 photoanode.

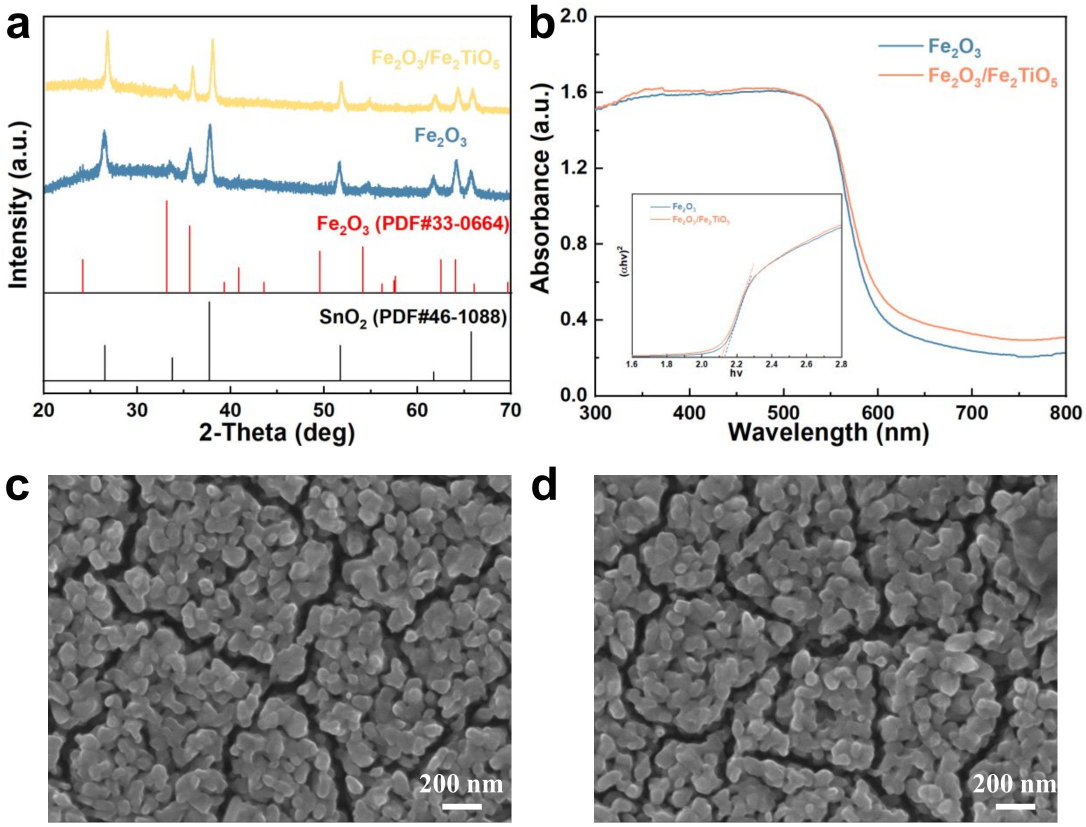

Figure S29. **a** XRD patterns and **b** UV-vis absorption spectra of Fe_2O_3 and Fe_2O_3/Fe_2TiO_5 photoanode. SEM imanges of **c** pristine Fe_2O_3 and **d** Fe_2O_3/Fe_2TiO_5 photoanode.

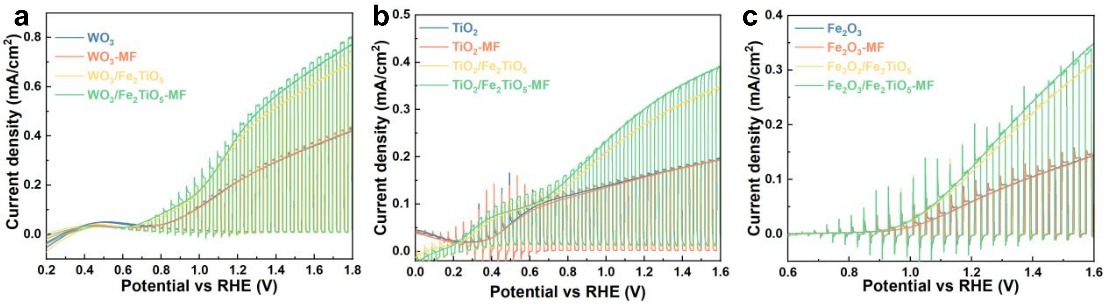

Figure S30. **a** LSV curves of **a** WO_3, WO_3/Fe_2TiO_5 photoanodes, **b** TiO_2 and TiO_2/Fe_2TiO_5 and **c** Fe_2O_3 and Fe_2O_3/Fe_2TiO_5 photoanode with or without external magnetic field.

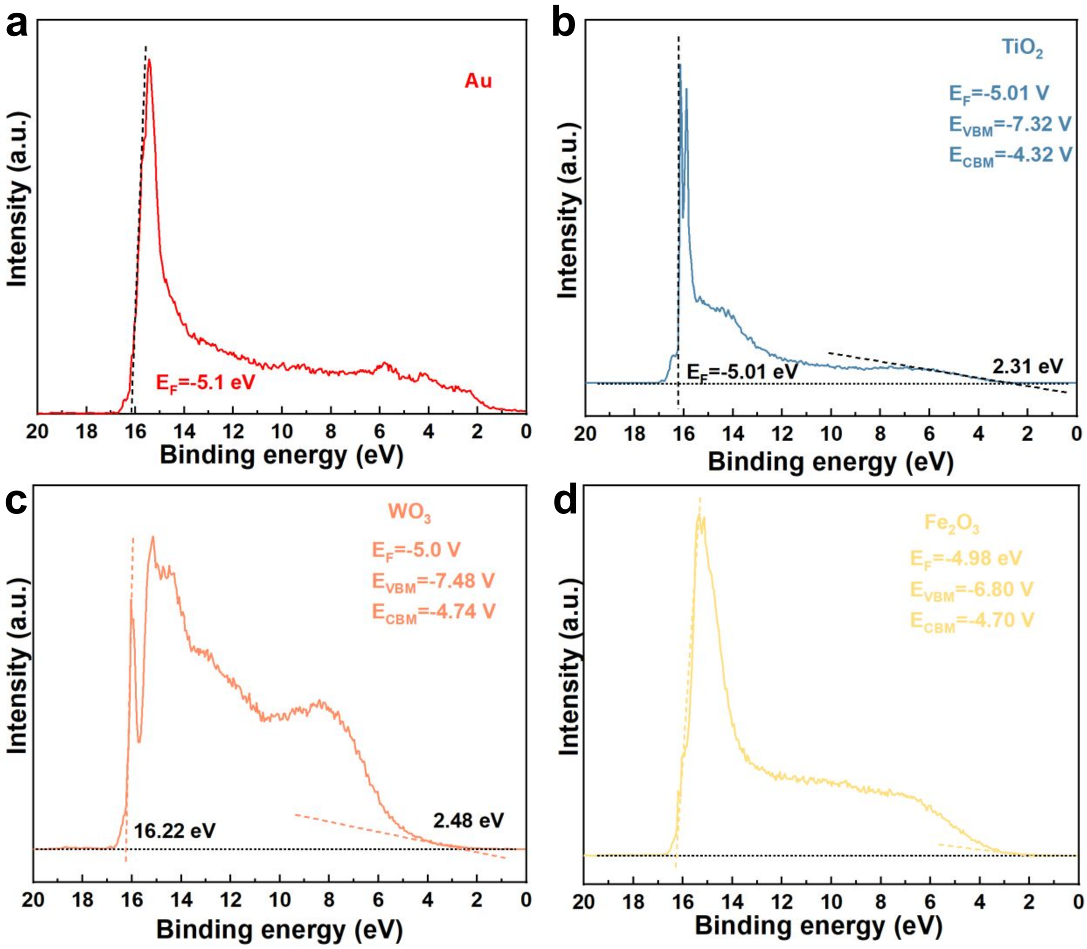

Figure 31. UPS spectra of **a** Au, **b** TiO_2, **c** WO_3 and **d** Fe_2O_3.

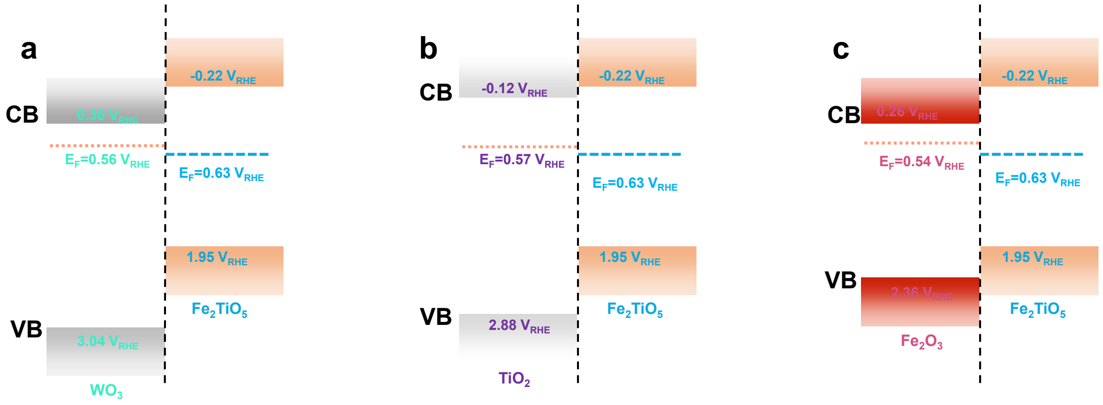

Figure S32. Energy band structure of **a** WO_3, **b** TiO_2, **c** Fe_2O_3 and Fe_2TiO_5 photoanode.

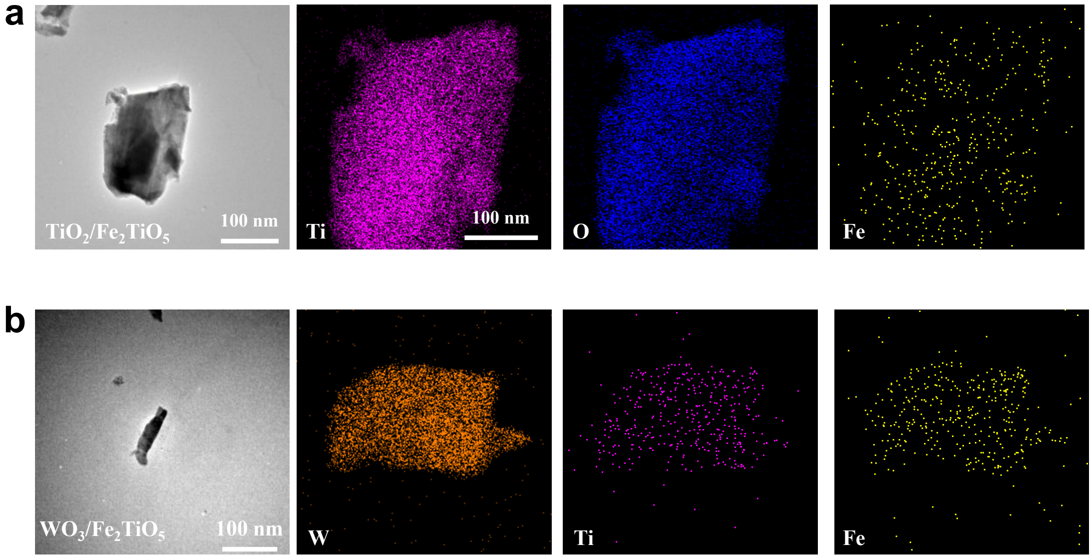

Figure S33. TEM images of **a** TiO_2/Fe_2TiO_5 and **b** WO_3/Fe_2TiO_5 with corresponding EDX mapping images.

Table S1. The standard quantitative elemental analysis obtained from the EDS elemental spectrum of the $BiVO_4/Fe_2TiO_5$ photoanode.

Element	*(KeV)*	*Mass%*	*Atom%*	*K*
Bi M	2.419	74.93	27.02	1.0000
V K	4.949	12.20	18.05	0.2950
O K	0.525	11.15	52.50	0.3841
Fe K	6.398	1.17	1.58	0.3353
Ti K	4.508	0.54	0.85	0.2795
Total		100	100	

Table S2. Comparison table for previously reported heterojunction photoanodes and the heterojunction photoanodes reported in this work for PEC water oxidation under AM 1.5G (100 mW·cm^{-2}) illumination.

Photoanode	Cocatalyst	HC-STH vs. RHE	J(mA/cm^2) 1.23 V vs. RHE	Stability	Reference
W:WO_3/$BiVO_4$	ND	ND	4.4	25 h	S6
$BiVO_4$/CoPy/PANI/CoFe	CoFe	1.3% (0.74V)	4.5	2 h	S7
IO-SnO_2/$BiVO_4$/FeOOH/NiOOH	FeOOH/NiOOH	1.02% (0.71V)	3.57	10 h	S8
$BiVO_4$/CTP	FeNiOOH	ND	4.05	10 h	S9
$BiVO_4$/Cu_2S/CoFe-OH	CoFe-OH	0.83% (0.69V)	3.07	10 min	S10
WO_3/$BiVO_4$/FeOOH/NiOOH	FeOOH/NiOOH	1.65% (0.72V)	5.5	5 h	S11
WO_3/$BiVO_4$	ND	ND	4.15	7 h	S12
FTO/TiO_2/$BiVO_4$/CoPi	CoPi	ND	4.11	ND	S13
$BiVO_4$/CoLa-LDH	CoLa-LDH	0.38% (0.61V)	2.1	ND	S14
$NiFeO_x$/CTF-BTh/Mo:$BiVO_4$	$NiFeO_x$	ND	5.7	150 h	S15
NiOOH/FeOOH/Co_3O_4/$BiVO_4$	NiOOH/FeOOH	2.72% (0.6V)	6.34	4 h	S16
OEC/MoO_x/MQD/$BiVO_4$	NiFeOOH	2.43 % (0.58 V)	5.85	100 h	S17
Co_3O_4/$BiVO_4$	ND	0.659% (0.82 V)	2.71	ND	S18
WO_3/S:Bi_2O_3/(Ga,W):$BiVO_4$/CoPi	CoPi	ND	5.1±0.25	10 h	S19
WO_3/(W, Mo)-$BiVO_4$	FeOOH/NiOOH	ND	5.35±0.15	84 h	S20
nanocone/Mo:$BiVO_4$/Fe(Ni)OOH	Fe(Ni)OOH	2.05% (0.62V)	5.82 ± 0.36	5 h	S21
3D TiO_2/WO_3/$BiVO_4$	FeOOH/NiOOH	ND	4.27	5 h	S22
In:GaN/Ta_3N_5/Mg:GaN	$CoNiFeO_x$	3.46% (0.75V)	9	160 min	S23
$NiFeO_x$/B-C_3N_4/Mo-$BiVO_4$	$NiFeO_x$	2.67% (0.54V)	5.93	10 h	S24
Fe_2O_3/Fe_2TiO_5	CoFe-PBA	ND	1.25	24 h	S25
ITO/ Fe_2O_3/Fe_2TiO_5/FeNiOOH	FeNiOOH	ND	2.2	7000 s	S26
TiO_2/Fe_2TiO_5/CoO_x	CoO_x	2.7% (0.42V)	2.9	2 h	S27
$BiVO_4$/Fe_2TiO_5	ND	1.15% (0.76 V)	3.03	6 h	**This work**
$BiVO_4$/Fe_2TiO_5/MF	ND	1.42% (0.70V)	3.33	6 h	**This work**

Reference

S1. Kim, T. W.; Choi, K.-S., Nanoporous $BiVO_4$ Photoanodes with Dual-Layer Oxygen Evolution Catalysts for Solar Water Splitting. *Science* **2014,** *343* (6174), 990-994.

S2. Tian, Z.; Da, Y.; MengWang; Dou, X.; Cui, X.; Chen, J.; Jiang, R.; Xi, S.; BaihuaCui; YaniLuo; Yang, H.; Long, Y.; Xiao, Y.; Chen, W., Selective photoelectrochemical oxidation of glucose to glucaric acid by single atom Pt decorated defective TiO_2. *Nature Communications* **2023,** *14* (1), 142-154.

S3. Li, Z.; Luo, L.; Li, M.; Chen, W.; Liu, Y.; Yang, J.; Xu, S. M.; Zhou, H.; Ma, L.; Xu, M.; Kong, X.; Duan, H., Photoelectrocatalytic C-H halogenation over an oxygen vacancy-rich TiO_2 photoanode. *Nat Commun* **2021,** *12* (1), 6698-6711.

S4. Deng, J.; Lv, X.; Nie, K.; Lv, X.; Sun, X.; Zhong, J., Lowering the Onset Potential of Fe_2TiO_5/Fe_2O_3 Photoanodes by Interface Structures: F- and Rh-Based Treatments. *ACS Catalysis* **2017,** *7* (6), 4062-4069.

S5. Wang, Q.; Wu, L.; Zhang, Z.; Cheng, J.; Chen, R.; Liu, Y.; Luo, J., Elucidating the Role of Hypophosphite Treatment in Enhancing the Performance of $BiVO_4$ Photoanode for Photoelectrochemical Water Oxidation. *ACS Appl Mater Interfaces* **2022,** *14*, 26642−26652.

S6. Liu, S.; Gao, R.-T.; Zhang, R.; Wang, Z.; Liu, X.; Nakajima, T.; Zhang, X.; Su, Y.; Wang, L., Tungsten induced defects control on $BiVO_4$ photoanodes for enhanced solar water splitting performance and photocorrosion resistance. *Applied Catalysis B: Environmental* **2021,** *298*, 120610.

S7. Yin, D.; Ning, X.; Du, P.; Zhang, D.; Zhang, Q.; Lu, X., Cascaded multiple-step hole transfer for enhancing photoelectrochemical water splitting. *Applied Catalysis B: Environmental* **2021,** *296*, 120313.

S8. Reddy, D. A.; Reddy, K. A. J.; Hong, D. H.; Gopannagari, M.; Kumar, D. P.; Kim, T. K., Constructing ordered paths to improve the charge separation and light harvesting capacity towards efficient solar water oxidation performance. *Applied Catalysis B: Environmental* **2020,** *269*, 118761.

S9. Pan, Q.; Chen, T.; Ma, L.; Wang, G.; Hu, W.-B.; Zou, Z.; Wen, K.; Yang, H., Covalent Triazine-Based Polymers with Controllable Band Alignment Matched with $BiVO_4$ To Boost Photogeneration of Holes for Water Splitting. *Chemistry of Materials* **2019,** *31* (19), 8062-8068.

S10. Zhu, S.-S.; Zhang, Y.; Zou, Y.; Guo, S.-Y.; Liu, H.; Wang, J.-J.; Braun, A., $Cu_2S/BiVO_4$ Heterostructure Photoanode with Extended Wavelength Range for Efficient Water Splitting. *The Journal of Physical Chemistry C* **2021,** *125* (29), 15890-15898.

S11. Zhou, Y.; Zhang, L.; Lin, L.; Wygant, B. R.; Liu, Y.; Zhu, Y.; Zheng, Y.; Mullins, C. B.; Zhao, Y.; Zhang, X.; Yu, G., Highly Efficient Photoelectrochemical Water Splitting from Hierarchical $WO_3/BiVO_4$ Nanoporous Sphere Arrays. *Nano Lett* **2017,** *17* (12), 8012-8017.

S12. Lee, B. R.; Lee, M. G.; Park, H.; Lee, T. H.; Lee, S. A.; Bhat, S. S. M.; Kim, C.; Lee, S.; Jang, H. W., All-Solution-Processed $WO_3/BiVO_4$ Core-Shell Nanorod Arrays for Highly Stable Photoanodes. *ACS Appl Mater Interfaces* **2019,** *11* (22), 20004-20012.

S13. Zhang, H.; Cheng, C., Three-Dimensional $FTO/TiO_2/BiVO_4$ Composite Inverse Opals Photoanode with Excellent Photoelectrochemical Performance. *ACS Energy Letters* **2017,** *2* (4), 813-821.

S14. Chhetri, M.; Dey, S.; Rao, C. N. R., Photoelectrochemical Oxygen Evolution Reaction Activity of Amorphous Co–La Double Hydroxide-$BiVO_4$ Fabricated by Pulse Plating

Electrodeposition. *ACS Energy Letters* **2017,** *2* (5), 1062-1069.
S15. Zhang, Y.; Lv, H.; Zhang, Z.; Wang, L.; Wu, X.; Xu, H., Stable Unbiased Photo-Electrochemical Overall Water Splitting Exceeding 3% Efficiency via Covalent Triazine Framework/Metal Oxide Hybrid Photoelectrodes. *Adv Mater* **2021,** *33* (15), e2008264.
S16. He, B.; Jia, S.; Zhao, M.; Wang, Y.; Chen, T.; Zhao, S.; Li, Z.; Lin, Z.; Zhao, Y.; Liu, X., General and Robust Photothermal-Heating-Enabled High-Efficiency Photoelectrochemical Water Splitting. *Adv Mater* **2021,** *33* (16), e2004406.
S17. Song, Y.; Zhang, X.; Zhang, Y.; Zhai, P.; Li, Z.; Jin, D.; Cao, J.; Wang, C.; Zhang, B.; Gao, J.; Sun, L.; Hou, J., Engineering MoO_x/MXene Hole Transfer Layers for Unexpected Boosting of Photoelectrochemical Water Oxidation. *Angew Chem Int Ed Engl* **2022,** *61* (16), e202200946.
S18. Chang, X.; Wang, T.; Zhang, P.; Zhang, J.; Li, A.; Gong, J., Enhanced Surface Reaction Kinetics and Charge Separation of p-n Heterojunction Co_3O_4/$BiVO_4$ Photoanodes. *J Am Chem Soc* **2015,** *137* (26), 8356-8359.
S19. Prasad, U.; Young, J. L.; Johnson, J. C.; McGott, D. L.; Gu, H.; Garfunkel, E.; Kannan, A. M., Enhancing interfacial charge transfer in a WO_3/$BiVO_4$ photoanode heterojunction through gallium and tungsten co-doping and a sulfur modified Bi2O3 interfacial layer. *Journal of Materials Chemistry A* **2021,** *9* (29), 16137-16149.
S20. Shi, X.; Choi, I. Y.; Zhang, K.; Kwon, J.; Kim, D. Y.; Lee, J. K.; Oh, S. H.; Kim, J. K.; Park, J. H., Efficient photoelectrochemical hydrogen production from bismuth vanadate-decorated tungsten trioxide helix nanostructures. *Nat Commun* **2014,** *5*, 4775-4783.
S21. Qiu, Y.; Liu, W.; Chen, W.; Zhou, G.; Hsu, P.-C.; Zhang, R.; Liang, Z.; Fan, S.; Zhang, Y.; Cui, Y., Efficient solar-driven water splitting by nanocone $BiVO_4$-perovskite tandem cells. *Sci advance* **2016,** *2*, e1501764.
S22. Zhang, H.; Zhou, W.; Yang, Y.; Cheng, C., 3D WO_3/$BiVO_4$/Cobalt Phosphate Composites Inverse Opal Photoanode for Efficient Photoelectrochemical Water Splitting. *Small* **2017,** *13* (16), 1603840.
S23. Fu, J.; Fan, Z.; Nakabayashi, M.; Ju, H.; Pastukhova, N.; Xiao, Y.; Feng, C.; Shibata, N.; Domen, K.; Li, Y., Interface engineering of Ta_3N_5 thin film photoanode for highly efficient photoelectrochemical water splitting. *Nat Commun* **2022,** *13* (1), 729-738.
S24. Ye, K. H.; Li, H.; Huang, D.; Xiao, S.; Qiu, W.; Li, M.; Hu, Y.; Mai, W.; Ji, H.; Yang, S., Enhancing photoelectrochemical water splitting by combining work function tuning and heterojunction engineering. *Nat Commun* **2019,** *10* (1), 3687-3696.
S25. Tang, P. Y.; Han, L. J.; Hegner, F. S.; Paciok, P.; Biset‐Peiró, M.; Du, H. C.; Wei, X. K.; Jin, L.; Xie, H. B.; Shi, Q.; Andreu, T.; Lira‐Cantú, M.; Heggen, M.; Dunin‐Borkowski, R. E.; López, N.; Galán‐Mascarós, J. R.; Morante, J. R.; Arbiol, J., Boosting Photoelectrochemical Water Oxidation of Hematite in Acidic Electrolytes by Surface State Modification. *Advanced Energy Materials* **2019,** *9* (34), 1901836.
S26. Tang, P.; Xie, H.; Ros, C.; Han, L.; Biset-Peiró, M.; He, Y.; Kramer, W.; Rodríguez, A. P.; Saucedo, E.; Galán-Mascarós, J. R.; Andreu, T.; Morante, J. R.; Arbiol, J., Enhanced photoelectrochemical water splitting of hematite multilayer nanowire photoanodes by tuning the surface state via bottom-up interfacial engineering. *Energy & Environmental Science* **2017,** *10* (10), 2124-2136.
S27. Liu, Q.; He, J.; Yao, T.; Sun, Z.; Cheng, W.; He, S.; Xie, Y.; Peng, Y.; Cheng, H.; Sun, Y.; Jiang, Y.; Hu, F.; Xie, Z.; Yan, W.; Pan, Z.; Wu, Z.; Wei, S., Aligned Fe_2TiO_5-containing

nanotube arrays with low onset potential for visible-light water oxidation. *Nat Commun* **2014,** *5*, 5122-5129.
